# Extreme mutation bias and high AT content in *Plasmodium falciparum*

**DOI:** 10.1093/nar/gkw1259

**Published:** 2016-12-19

**Authors:** William L. Hamilton, Antoine Claessens, Thomas D. Otto, Mihir Kekre, Rick M. Fairhurst, Julian C. Rayner, Dominic Kwiatkowski

**Affiliations:** 1 Malaria Programme, Wellcome Trust Sanger Institute, Hinxton, CB10 1SA, UK; 2 University of Cambridge School of Clinical Medicine, Cambridge Biomedical Campus, Hills Road, Cambridge, CB2 0SP, UK; 3 Medical Research Council Unit The Gambia, Atlantic Road, Fajara, P.O. Box 273, Banjul, The Gambia; 4 Department of Pathogen Molecular Biology, London School of Hygiene and Tropical Medicine, London, WC1E 7HT, UK; 5 Laboratory of Malaria and Vector Research, National Institute of Allergy and Infectious Diseases, National Institutes of Health, Rockville, Maryland, USA; 6 Wellcome Trust Centre for Human Genetics, University of Oxford, Oxford, OX3 7BN, UK

## Abstract

For reasons that remain unknown, the *Plasmodium falciparum* genome has an exceptionally high AT content compared to other *Plasmodium* species and eukaryotes in general - nearly 80% in coding regions and approaching 90% in non-coding regions. Here, we examine how this phenomenon relates to genome-wide patterns of *de novo* mutation. Mutation accumulation experiments were performed by sequential cloning of six *P. falciparum* isolates growing in human erythrocytes *in vitro* for 4 years, with 279 clones sampled for whole genome sequencing at different time points. Genome sequence analysis of these samples revealed a significant excess of G:C to A:T transitions compared to other types of nucleotide substitution, which would naturally cause AT content to equilibrate close to the level seen across the *P. falciparum* reference genome (80.6% AT). These data also uncover an extremely high rate of small indel mutation relative to other species, primarily associated with repetitive AT-rich sequences, in addition to larger-scale structural rearrangements focused in antigen-coding *var* genes. In conclusion, high AT content in *P. falciparum* is driven by a systematic mutational bias and ultimately leads to an unusual level of microstructural plasticity, raising the question of whether this contributes to adaptive evolution.

## INTRODUCTION

The malaria parasite *Plasmodium falciparum* afflicts hundreds of millions of people worldwide and kills half a million African children each year. One of the major obstacles to controlling malaria is the parasite's prolific capacity for evolutionary adaptation. By a continual process of mutation and recombination, the parasite is able to generate very large numbers of new antigenic variants to evade the human immune system, thus complicating vaccine development, and to develop resistance to all drugs that are widely deployed as frontline antimalarial therapy. The most recent example of this phenomenon is the epidemic of artemisinin resistance that is spreading through Southeast Asia and threatening to destabilise malaria control worldwide (1).

**Figure 1.**
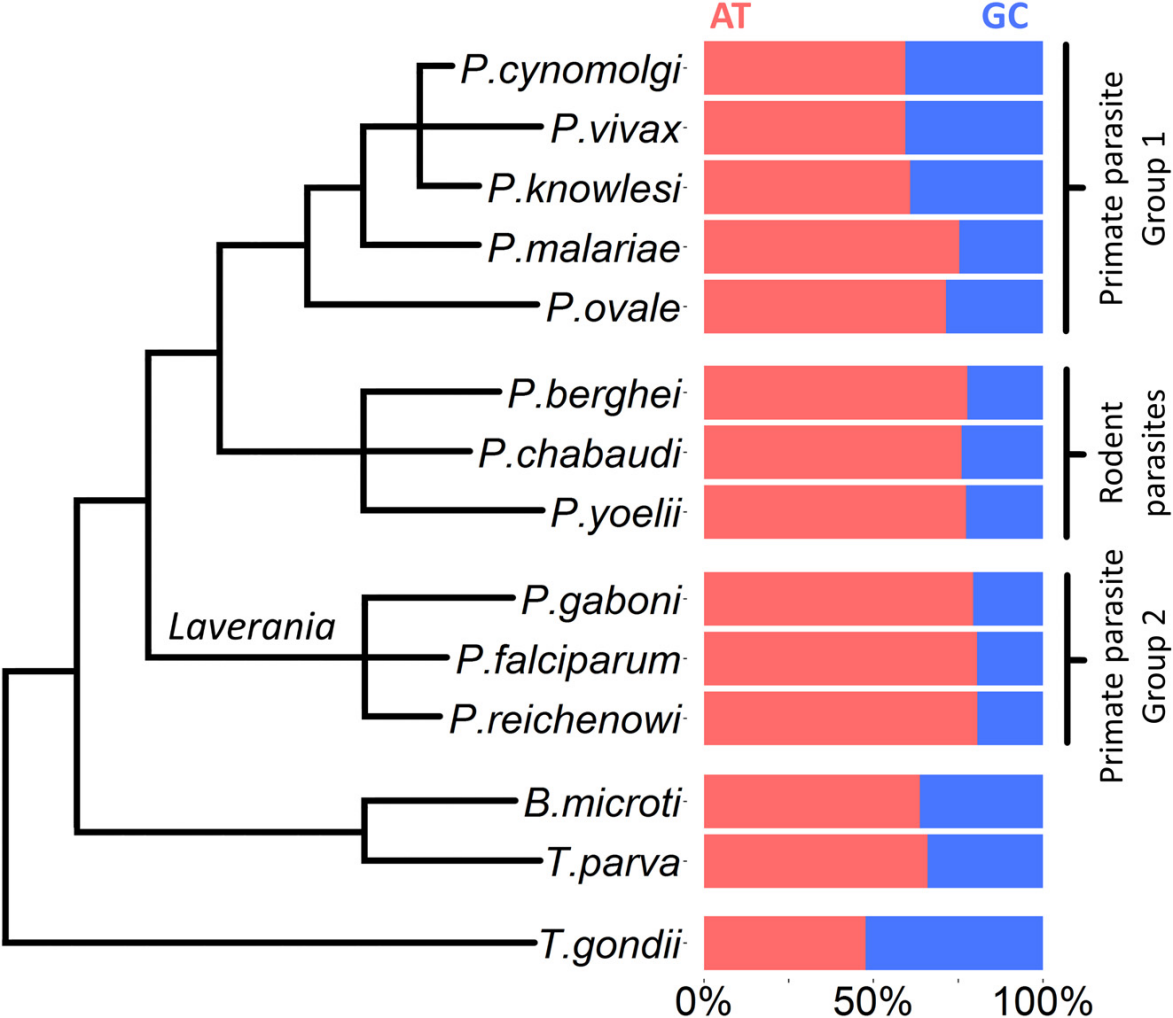
AT content variation in Apicomplexan parasites. Phylogenetic relationships and AT:GC genome proportions are shown for a range of Apicomplexan parasite species. Phylogenetic lines are not to evolutionary scale. AT contents are derived from NCBI genome browser data (http://www.ncbi.nlm.nih.gov/genome/) and shown in Supplementary Table S1. Phylogeny of *Plasmodium* parasites is based on (42), Figure 1a. Phylogeny of the non-*Plasmodium* parasites *Theileria parva, Babesia microti* and *Toxoplasma gondii* is based on (44).

Despite a large scientific literature on genetic diversity in *P. falciparum*, there remain important gaps in our understanding of the processes of mutation and recombination that underlie the parasite's ability to adapt rapidly to new evolutionary pressures. A question of fundamental interest is the role played by ongoing mutational processes in shaping the parasite's unusual genome architecture, particularly its exceptionally high AT content (80.6% AT in the 3D7 isolate), one of the most skewed base pair compositions of any eukaryote (2,3), and its expanses of repetitive, low-complexity regions, which are widely dispersed throughout the genome (4,5). For comparison, the AT contents of *Homo sapiens, Drosophila melanogaster, Arabidopsis thaliana* and the eukaryotic pathogens *Toxoplasma gondii* and *Trypanosoma bruceii* are 58.9%, 57.9%, 63.4%, 47.7% and 53.2%, respectively [NCBI Genome database: http://www.ncbi.nlm.nih.gov/genome/] (Supplementary Table S1). The *Plasmodium* genus as a whole exhibits high AT content, with the highest levels seen in *P. falciparum* and the related *Laverania* parasites *P. reichenowi* and *P. gaboni*, while *P. vivax* has a more even genome composition at 59.4% (Figure 1). The biological causes of these differences are currently unknown.

We investigated the mutational origins and consequences of high AT content using an experimental evolution approach in which *P. falciparum* parasites were cultured *in vitro* for hundreds of erythrocytic life cycles and *de novo* mutations were detected by whole genome sequencing. Mutation accumulation experiments, or ‘clone trees’, have previously been reported in *P. falciparum* using established long-term culture-adapted laboratory isolates (6,7). Here, we have enlarged the scale of these experiments by analysing six clone trees, including recently culture-adapted field isolates from Western Cambodia, producing the largest dataset of *de novo* mutation in *P. falciparum* published to date. This allowed us to calculate mutation rates for each type of base pair substitution (BPS, the mutation origin of single nucleotide polymorphisms (SNP) detected in population genetic data), and so quantify how ongoing mutational biases contribute to maintaining *P. falciparum*'s skewed base pair composition. We then analysed what impact the parasite's AT-rich, repetitive DNA sequences has on the rate of spontaneous insertion/deletion (indel) mutations, demonstrating a prolifically high indel rate, over ten-fold higher than the BPS rate. We discuss the potential causes and biological significance of this BPS bias, AT-richness and high indel mutation rate.

## MATERIALS AND METHODS

### Parasite *in vitro* culture and clone tree generation

**Figure 2.**
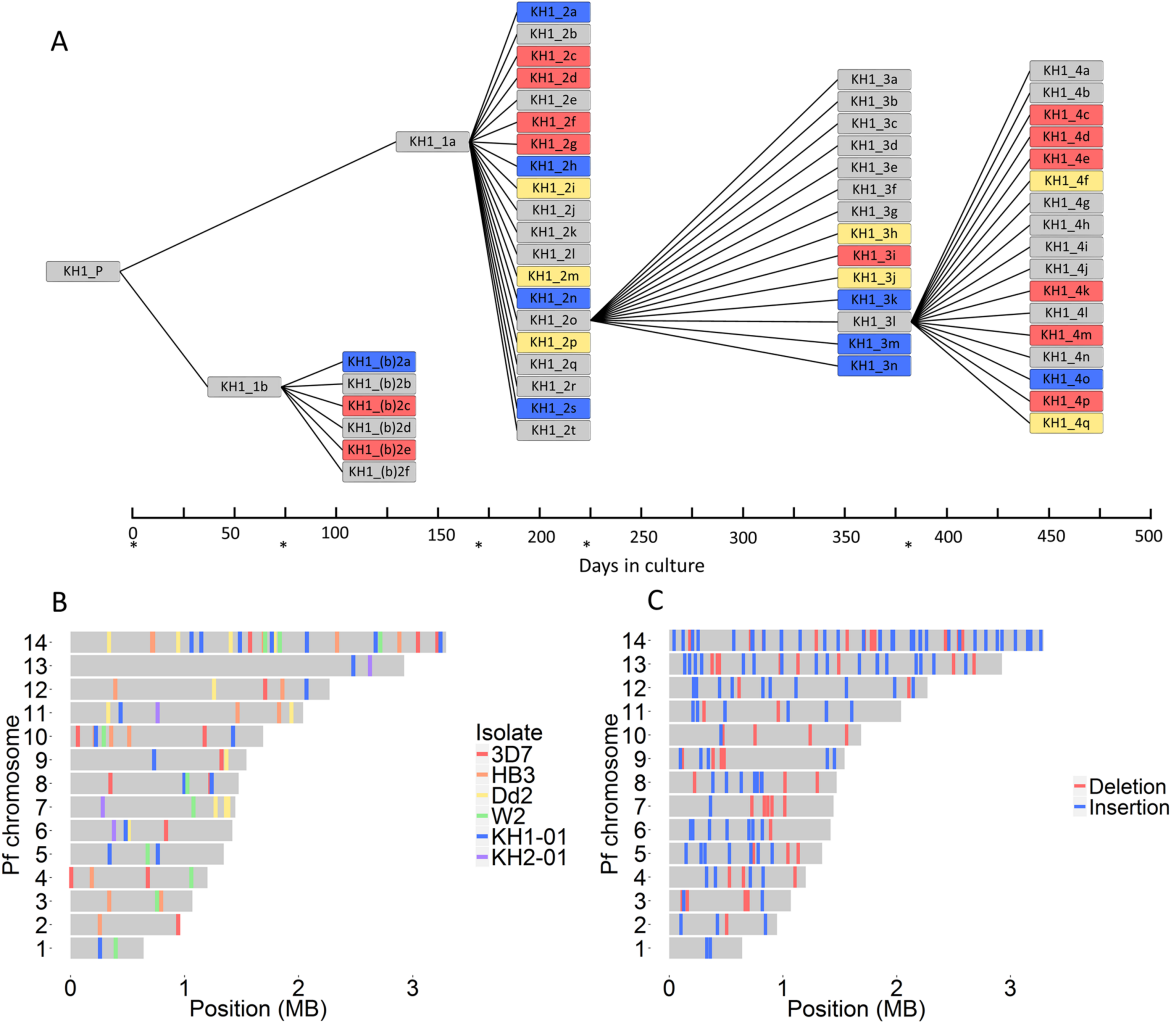
Detecting *de novo* mutations in mitotic ‘clone trees’. (**A**) Schematic of the KH1-01 clone tree. Each branch point represents a round of subcloning by limiting dilution, isolating single infected red blood cells, and asexually expanding them until we obtained sufficient amounts of DNA for whole-genome sequencing. The KH1-01 clone tree was cultured for ∼17 months and contained 59 subclone genomes derived from the parent isolate (obtained soon after venous sampling from a patient in Cambodia). Blue = *de novo* BPS detected; red = pairs of recombining *var* genes detected; yellow = both BPS and *var* gene recombination detected; asterisks = clonal dilutions performed. (**B**) Overall, in the clone trees of six wild-type *P. falciparum* isolates (3D7, HB3, Dd2, W2, KH1-01 and KH2-01), we identified 85 *de novo* BPS, 78 of which have known genomic coordinates (shown here). The 78 BPS with known genomic coordinates were distributed across the 14 nuclear chromosomes as expected by chance (*P* = 0.445, Fisher's exact test). Colors depict the isolate in which the BPS occurred. (**C**) We identified 164 *de novo* indels in the 3D7 clone tree, all of which have known genomic coordinates (shown here). Like BPS, indel distribution between the 14 chromosomes did not differ significantly from what is expected by chance (*P* = 0.194, Pearson's Chi-squared test). Insertions (*n* = 108) and deletions (*n* = 56) are colored blue and red, respectively.

To analyse patterns of *de novo* mutation, we generated six mutation accumulation lines using the same approach previously reported (6). Briefly, we cultured parasites continuously *in vitro* with periodic subcloning to isolate single infected red blood cells, which were asexually expanded to provide DNA for whole genome Illumina sequencing. After 1–4 months of continuous culture, we selected one of these subclones for another round of cloning by limiting dilution. We refer to each of these cycles as clone tree ‘generations’ (Figure 2A). One advantage of this approach is that it reduces the impact of selection, thus approximating the molecular mutation rate (8).

All parasites were cultured in human O+ erythrocytes with heat-inactivated 10% pooled human serum as described in (9). Clone tree production in laboratory isolates (3D7, HB3, Dd2, W2) has been described previously, and the genomes from these clone trees were published in (6). We used the same method to produce clone trees from recently culture-adapted Cambodian field isolates, referred to as ‘KH1-01’ and ‘KH2-01’. Parasites were ultra-diluted into a 96-well plate, to reach a theoretical concentration of 0.2–0.5 parasites per well. We identified positive wells microscopically and further cultured parasites in a 10-ml flask, before extracting DNA and producing glycerolyte frozen stocks. One subclone was arbitrarily selected for the next round of clonal dilution to produce the next clone tree ‘generation’ (Figure 2 and Supplementary Figure S1). Median culture time between clonal dilution rounds was 49.5 days (range: 29–165; interquartile range (IQR): 28). We submitted ∼1–2 ug DNA for PCR-free library preparation by the Wellcome Trust Sanger Institute core sequencing teams for paired-end sequencing on the Illumina HiSeq 2000 platform. Conditions were as described in (10), except without the initial PCR amplification step. We selected DNA fragments with an insert size of ∼400 bp, producing paired-end reads of 100 bp each.

KH1-01 and KH2-01 parasites from Western Cambodia were frozen soon after patients’ sampling. KH2-01 had prolonged *in vivo* clearance following artesunate therapy (half-life: 10.08 hours), while KH1-01 had rapid clearance typical of artemisinin-sensitive parasites (half-life: 3.51 h). The isolates cluster within the KH1 and KH2 subpopulations defined by Miotto *et al*. (11), and KH2-01 carries the *kelch13* C580Y mutation associated with artemisinin resistance, while KH1-01 is *kelch13* wild-type. KH2-01 also possessed several of the highly differentiated SNPs in DNA repair genes identified by Miotto *et al.* (11), including P203S in *mlh1*, S506I in *pms1*, and C2010S in *rad50*.

### Genome mapping

We processed all samples through the MalariaGEN pipeline, as described (6,12). Briefly, we mapped BAM files to the *P. falciparum* 3D7 reference genome (version 3) using BWA with default parameters. For the discovery of translocations within KH1-01 and KH2-01 *var* genes, we also mapped sequences to an assembled library of *var* gene sequences from KH1-01 and KH2-01 (ENA Accession numbers are in Supplementary Table S2).

Since we had no reference for the Cambodian isolates and we mapped their sequences to the 3D7 genome, we note that there may be a greater proportion of false negatives (i.e. missed mutations) in the Cambodian parasites. However, this is unlikely to dramatically affect the mutation rate, as the proportion of the 3D7 reference genome with ≥5× coverage was only slightly lower in the KH1-01 and KH2-01 subclone genomes (median 95.6%, IQR 4.7% versus 94.7%, IQR 2.1%, respectively) compared with the 3D7 subclone genomes (median 99.4%, IQR 2.5%) (difference between KH1-01 v 3D7 and KH2-01 v 3D7 both statistically significant: *P* = 8.96 × 10^−9^ and *P* = 1.94 × 10^−6^, respectively, two-sample Wilcoxon rank sum tests (discussed further in Supplementary Appendix S1)).

### Base pair substitution calling

We used SAMtools mpileup followed by BCFtools (13) to detect BPS, exactly as described previously (6), and confirmed all variants by manual inspection on LookSeq (14). As expected, larger clone trees (with more subclone genomes analysed and longer *in vitro* culture times) yielded more *de novo* BPS than smaller ones (Supplementary Figure S2).

### Indel calling

We used Genome Analysis Toolkit (GATK) software (version 3.3.0) (15) to call indels, as in (16). Using a known set of indels from the progeny of genetic crosses (3D7xHB3, Dd2xHB3, and 7G8xGB4) (16), we realigned each 3D7 BAM separately using options ‘RealignTargetCreator’ followed by ‘IndelRealigner.’ We then used the ‘UnifiedGenotyper,’ with ploidy = 1, to call indels in all re-aligned BAM files simultaneously. We used ‘VQSR’ to recalibrate indels and generate a VQSlod score (log odds ratio of being a true variant versus being false) for each indel. A list of indel hits found solely in a progeny sample and not its parent was generated and low-quality hits were filtered out if they failed to pass any of following parameters: Read counts ≥6, Genotype Quality ≥50, Phred-scale Likelihood ≥100, (Alternate reads/total reads) >0.6, VQSLOD >0. We visually inspected all 180 hits on LookSeq (14), and manually filtered out 16 hits (8.9%), as they could be sequencing/mapping errors (Supplementary Figure S3).

### Structural variant calling

We called structural variants in *var* genes using DELLY (17) with default parameters as described (6). We used reads with one end mapped to a chromosome and the second end mapped to another to identify translocations, with a minimum cut-off of five reads. As with BPS, only structural variants found uniquely in progeny VCF files and not in the parent progressed to further analysis, thus identifying only *de novo* mutations. We manually inspected all resulting hits on LookSeq (14). We mapped reads from each sample with BWA to separate *var* gene sequences, i.e. each *var* sequence was considered its own ‘chromosome.’

### Variant validation

The BPS validation process we used has been previously reported (6). Briefly, a 150–800 bp fragment containing the putative mutation is PCR amplified and capillary sequenced from wild-type controls and the sample containing the putative mutation, with sequences aligned and visualized using ClustalOmega and ClustalX. Primers were designed using Primer3 software (all primer sequences in Supplementary Table S11). We attempted to validate 17 indels from the 3D7 Illumina data using a similar approach, but only eight of these produced interpretable capillary sequence data (Supplementary Table S10) owing to the indels occurring in regions that were very difficult to PCR amplify (highly repetitive and AT rich). Eight out of eight indels called by Illumina were supported by the capillary sequence data.

Chimeric *var* genes identified by DELLY were also validated through PCR as described in (6), by designing primers that bridged the putative translocation sites and only amplified product in samples possessing that translocation, or amplified products of different sizes in wild-type and mutant parasites. In addition to the validations presented in (6), we used PCR amplification and capillary sequencing to validate the chimeric *CARvar15-76* translocation in subclone KH2_2s from the KH2-01 clone tree.

### Calculating the BPS, indel and *var* exon 1 recombination rates

We calculated mutation rates using the same methodology as in (6). We calculated separate mutation rates (μ_1_) per erythrocytic life cycle (ELC) for each clone tree generation using the number of *de novo* mutations (*s*) identified per subclone genome analysed within that generation (*c*), divided by the number of erythrocyte life cycles (*L*) since the previous clonal dilution (Equation 1). To derive mutation rates per ELC per base pair (μ_2_), we divided μ_1_ by the total genome size of *P. falciparum* (*G*), 2.33 × 10^7^ bp (Equation 2).

(1) \begin{equation*}{\mu _1} = \frac{{\left( {\frac{{\mathop \sum \nolimits s}}{{\mathop \sum \nolimits c}}} \right)}}{L}\ \end{equation*}

(2) \begin{equation*}{\mu _2} = \frac{{\left( {\frac{{\mathop \sum \nolimits s}}{{\mathop \sum \nolimits c}}} \right)}}{{L*G}}\ = \frac{{{\mu _1}}}{G}\ \end{equation*}

The mutation rates for each isolate and for all isolates were then derived by taking the mean of the rates in each clone tree generation, with standard deviations and 95% confidence intervals. The mean was weighted by the size of each clone tree generation (*w*), described in Equation (3).

(3) \begin{equation*}w\ = \ dc\end{equation*}

where *w* is the weighting factor for that clone tree generation, *d* is total days of *in vitro* culture for that generation and *c* is number of subclone genomes analyzed in that generation. We chose this weighting because it had the best correlation with number of *de novo* BPS identified across the six clone trees (*r*^2^ = 0.908, *P* = 0.00211, linear regression), above *d* or *c* individually (Supplementary Figure S2).

**Figure 3.**
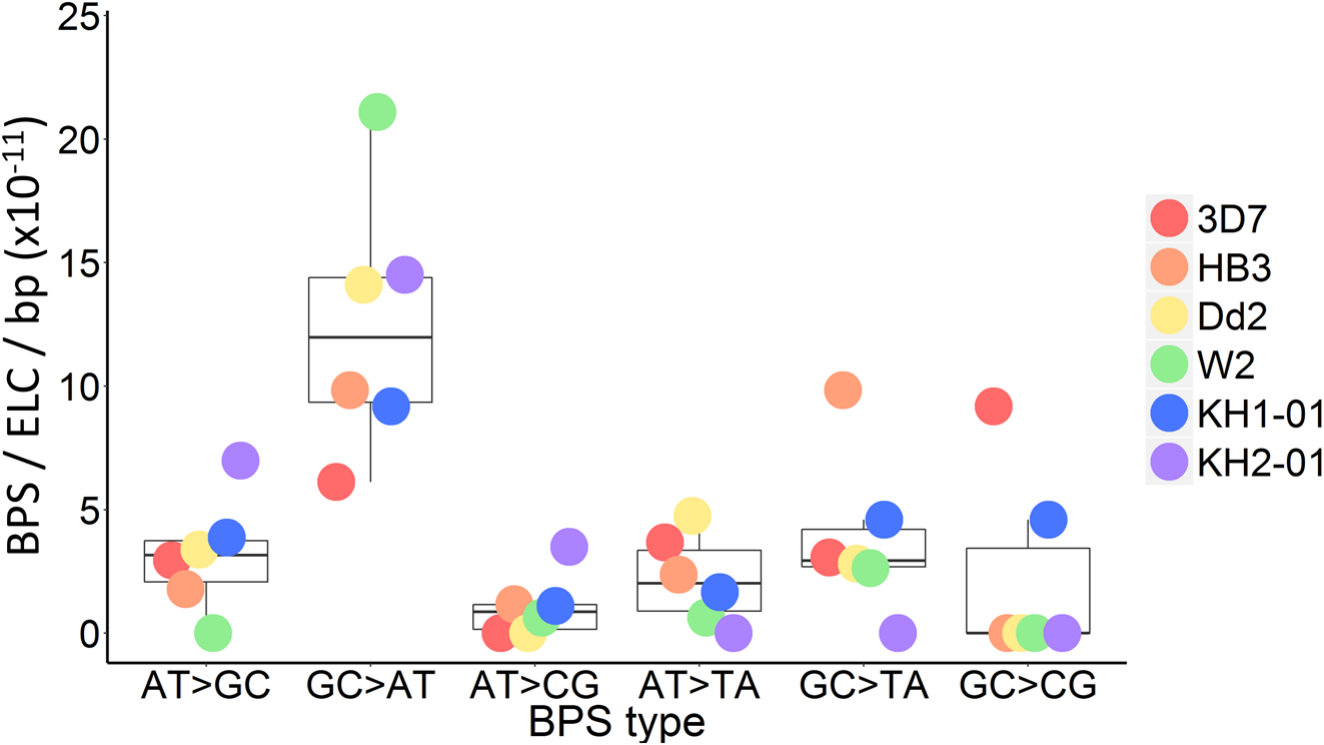
Base pair substitution spectrum across all isolates. Boxplots show the median rate of each base pair substitution (BPS) type with first and third quartiles, while coloured dots indicate that value for each isolate. ELC = erythrocytic life cycle; bp = base pair.

To calculate BPS rates for each substitution type, the raw numbers of BPS in each type (A:T→G:C, G:C→A:T, A:T→C:G, A:T→T:A, G:C→T:A, and G:C→C:G) were divided by the total number of A/T or G/C nucleotides in the *P. falciparum* genome, taken as 18 779 800 bp and 4 520 200 bp, respectively (based on GC content of 19.36%), giving BPS/bp figures for each substitution type. These were then distributed between the mean overall BPS rate per ELC per bp for all clone trees (2.45 × 10^−10^ BPS/ELC/bp) to derive the BPS rates per ELC per bp for each substitution type, shown in Figure 3. (We use a colon to denote base pairing between complementary nucleobases on two DNA strands, and a slash to signify ‘either/or’ nucleotides on the same strand, e.g. A:T is a complementary base pairing whereas A/T can be either an adenine or a thymine.)

### Statistical methods

All statistical tests were performed using the programming language R. Datasets were assessed for normality by Shapiro–Wilk testing with α-level = 0.05. Normally distributed data were described with mean, standard deviation, and 95% Confidence Intervals, with two-sample Welch *t*-test for comparison between means with unequal variances and sample sizes. Bonferroni correction method was applied to *P*-values as stated in the text to account for multiple comparisons (18). Non-normal datasets were described with median and interquartile range, and compared via two-sample Wilcoxon rank sum test. Weighted Welch *t*-tests were performed using the R package *weights*.

To calculate statistical significance for BPS spectra, we took the raw BPS counts from the clone trees for the six different BPS types as the observed data. The ‘expected’ data assumed a null hypothesis in which all BPS types were equally likely, taking account of the skewed underlying A/T: G/C ratios in the *P. falciparum* genome. Observed vs expected data tables were compared using χ^2^ test. Indel size divisibility was assessed under the null hypothesis whereby indels would be of even length half the time, and divisible by three one third of the time. Further detail on contingency tables used for statistical tests is shown in Supplementary Appendix S2. Plots were created in R using *ggplot2* and *tidyr* packages. 95% Clopper-Pearson confidence intervals for binomial data were generated using the package *binom*.

### *Var* gene expression analysis

To analyse *var* gene expression, parasites in 10-ml flasks at 4% haematocrit were synchronized using 5% sorbitol and spun down at a ring-stage parasitaemia ≥4%. Parasites were stored in Ambion^®^ RNAlater^®^ Stabilization Solution (AM7024) at −4°C for a maximum of 48 h before RNA extraction with the Ambion® RiboPure™ RNA Purification Kit (AM1924) per the manufacturer's instructions. Stock RNA was stored at −80°C. cDNA was generated using the Applied Biosystems^®^ High-Capacity cDNA Reverse Transcription Kit (4368814) per the manufacturer's instructions. Negative controls were included in the cDNA reaction step (with no reverse transcriptase enzyme added) to indicate DNA contamination. Reaction conditions were as follows: 10 min 25°C/120 min 37°C/5 min 85°C. cDNA test samples and negative controls were used as template for a PCR using DBLα ‘universal primers’ (19,20). The Platinum® Pfx DNA polymerase kit was used with reaction conditions per manufacturer's instructions and an annealing temperature of 42°C. Samples of the PCR reactions were run on a 1% agarose gel, and the remainder was used for blunt-end cloning into the Invitrogen pCR vector and plating on kanamycin–agar plates. The specialist and small faculty sequencing team at the Sanger Institute received the bacterial plates for each of the Dd2 and KH1-01 subclones tested. A median of 96 and 45 colonies were submitted for sequencing in Dd2 and KH1-01, respectively. Sequence tags were identified using BLAST and ClustalW, with the Dd2 and KH1-01 *var* genes.

## RESULTS

### Clone trees

We analysed data from six *in vitro* mutation accumulation lines, or ‘clone trees’: four laboratory isolates (3D7, HB3, Dd2 and W2, previously reported on for analysis of antigen-coding genetic recombination (6)) and the recently culture-adapted field isolates from Western Cambodia, KH1-01 and KH2-01. In total, we analysed 279 whole genomes from subclones cultured for 1404 days *in vitro* (∼700 asexual erythrocytic life cycles) (Table 1, Supplementary Figure S1). After mapping against the 3D7 reference genome, the median coverage with at least five reads of all 279 genomes was 94.7% (interquartile range, IQR, 2.30%) of the genome covered, and no individual subclone was <80% covered (Supplementary Table S2).

**Table 1.** Base pair substitution counts and mutation rates in the clone trees

Isolate	No. subclones sequenced	Days in culture	BPS	BPS/ELC mean (×10^−3^)	BPS/ELC 95% CI (×10^−3^)	BPS/ELC/bp mean (×10^−10^)	BPS/ELC/bp 95% CI (×10^−10^)
3D7	37	203	15	4.91	3.48–6.33	2.10	1.49–2.72
HB3	81	250	17	6.88	3.30–10.45	2.95	1.42–4.49
Dd2	56	298	18	7.45	3.80–11.11	3.20	1.63–4.77
W2	20	119	11	N/A	N/A	N/A	N/A
KH1-01	59	447	20	5.29	1.29–9.28	2.27	0.550–3.98
KH2-01	26	87	4	3.83	0.330–7.34	1.64	0.142–3.15
Total	279	1404	85	5.71	3.96–7.46	2.45	1.70–3.20

The mean base pair substitution (BPS) rate of all generations of each isolate's clone tree, along with 95% confidence interval (CI), are shown. All means were weighted by the size of the clone tree generated, defined as the product of the number of subclone genomes analysed and number of days in culture (see Materials and Methods and Supplementary Figure S2). ELC = erythrocytic life cycle. We excluded W2 data from mutation rate calculations due to an insufficient number of BPS observed after the first generation of the clone tree.

### Biased nucleotide substitution rates drive AT-richness

We investigated whether biased BPS rates could explain the parasite's skewed genomic base pair composition. We identified 85 *de novo* nuclear BPS across all clone trees, plus one apicoplast BPS (Supplementary Table S3, Figure 2). Of the six possible BPS types, two are transitions (A:T→G:C and G:C→A:T) and four are transversions (A:T→C:G, A:T→T:A, G:C→T:A, and G:C→C:G). Hence, if all BPS were equally probable, the default transition-to-transversion ratio (Ts:Tv) would be 0.5. This differs significantly from the Ts:Tv ratio of 1.13 actually observed (Table 2) (*P* = 2.03 × 10^−4^, two-sided exact binomial test). This transition bias was mainly driven by an excess of G:C→A:T substitutions observed in all isolates (Figure 3). The mean G:C→A:T mutation rate per bp was higher than for any other substitution type (*P* < 0.01 for all pairwise comparisons, two-sample Welch *t*-test), while no other BPS type differed significantly from any other (Supplementary Table S4). The overall substitution spectrum across the six BPS types differed significantly from what would be expected if all substitutions occurred at an equal rate per bp (*P* = 5.13 × 10^-4^, Pearson's Chi-squared test).

**Table 2.** Raw base pair substitution counts in *P. falciparum* mutation-accumulation lines

Isolate	A:T→G:C	G:C→A:T	Ts	A:T→C:G	A:T→T:A	G:C→T:A	G:C→C:G	Tv	Ts:Tv
3D7	4	2	**6**	0	5	1	3	**9**	**0.67**
HB3	3	4	**7**	2	4	4	0	**10**	**0.70**
Dd2	5	5	**10**	0	7	1	0	**8**	**1.25**
W2	0	8	**8**	1	1	1	0	**3**	**2.67**
KH1-01	7	4	**11**	2	3	2	2	**9**	**1.22**
KH2-01	2	1	**3**	1	0	0	0	**1**	**3.00**
Clone tree	21	24	**45**	6	20	9	5	**40**	**1.13**
Genetic crosses	8	8	**16**	4	6	4	2	**16**	**1.00**
Total	29	32	**61**	10	26	13	7	**56**	**1.09**

The 3D7, HB3, Dd2, W2, KH1-01, and KH2-01 data are from clone tree experiments. ‘Clone tree’ shows the total for the six isolate clone trees. The ‘genetic crosses’ data are pooled *de novo* base pair substitutions from the progeny of three experimental genetic crosses: HB3xDd2, HB3×3D7, and 7G8xGB4. ‘Total’ shows combined counts for clone trees and genetic crosses. The overall Ts:Tv (Transition:Transversion) ratio for wild-type *P. falciparum* was 1.13, indicating transition bias. Every wild-type isolate had some degree of transition bias (i.e. Ts:Tv > 0.5), Ts = Transition; Tv = Transversion.

To increase the sample size, we analysed a set of 32 BPS identified in the progeny of three experimental genetic crosses: HB3xDd2, HB3×3D7 and 7G8xGB4 (16) (Table 2, Supplementary Table S5 and Figure S4). These isolates have been through many rounds of mitotic erythrocytic division before and after the crosses, and one round of meiotic division within mosquitoes. As with the clone trees, G:C→A:T mutations were the most common BPS per bp (Table 2, Supplementary Figure S4). The distribution of substitutions between the six BPS types did not differ significantly between the 85 clone tree BPS and 32 crosses BPS (*P* = 0.943, Pearson's Chi-squared test).

If the genome were at mutational equilibrium, the number of A/T conversions to G/C would equal G/C conversions to A/T, giving an expected ratio of 0.5. Our observed ratio (39:45, respectively) did not differ significantly from this (*P =* 0.586, two-sided exact binomial test). The total ratio of A/T→G/C versus G/C→A/T substitution rates per bp was 4.79, i.e. for every one A/T→G/C substitution there would be about five G/C→A/T substitutions, if the genome had an even base pair composition. We would expect this to equilibrate at a genomic base pair composition of ∼82.7% AT, close to the actual genomic average in *P. falciparum* (80.6% AT overall in the 3D7 reference genome). These data demonstrate that the parasite's high AT content is being maintained at or near mutational equilibrium by a skewed BPS spectrum.

We analyzed the pattern of nucleotides surrounding C→T transition sites for evidence of the underlying mechanism. C→T transitions commonly result from 5-methylcytosine deamination to thymine. Cytosine methylation is associated with CpG sites in mammalian genomes, though Ponts *et al.* have reported an enrichment for thymines surrounding conserved 5-methylcytosines in *P. falciparum* (21). We did not find any significant overrepresentation of any nucleotide in the +1 or −1 positions of our 24 G:C→A:T clone tree transitions compared with what would be expected by chance (Fisher's exact test). Another cause of G:C→A:T mutation is pyrimidine dimerisation, which can cause CpC→TpT transitions. We did not observe any instances of CpC→TpT mutations. However, with only 24 observations, our statistical power is low.

### AT-rich repetitive sequences undergo prolific rates of indel mutation

The AT-rich *P. falciparum* genome contains stretches of highly repetitive, low complexity DNA. We investigated whether this predisposes to a raised rate of indel mutation. We limited this analysis to the nuclear 3D7 genome, as other isolates would have yielded a significant ‘false negative’ rate (i.e. missed indels) due to the lack of a near-perfect reference genome (unlike 3D7), particularly in telomeric and subtelomeric loci. We identified 164 *de novo* indels in 37 subclone genomes in 3D7, compared with 15 BPS (Figure 2C, Supplementary Table S6). There were 108 insertions and 56 deletions, with median insertion and deletion sizes of 2 and 8 bp, respectively (size difference between insertions and deletions was statistically significant, *P* = 6.01 × 10^-9^, two-sample Wilcoxon rank sum test). The 3D7 indel rate was extremely high, around 10-fold greater than the BPS rate, at 21.1 × 10^−10^ (95% confidence interval, CI, 13.2 × 10^−10^–28.9 × 10^−10^) indels/ELC/bp; i.e. about one in every 20 parasites (95%CI 15–33) would be expected to harbor a *de novo* indel per 48-h ELC, compared with one *de novo* BPS in every 175 parasites (95%CI 134–253) per ELC.

**Figure 4.**
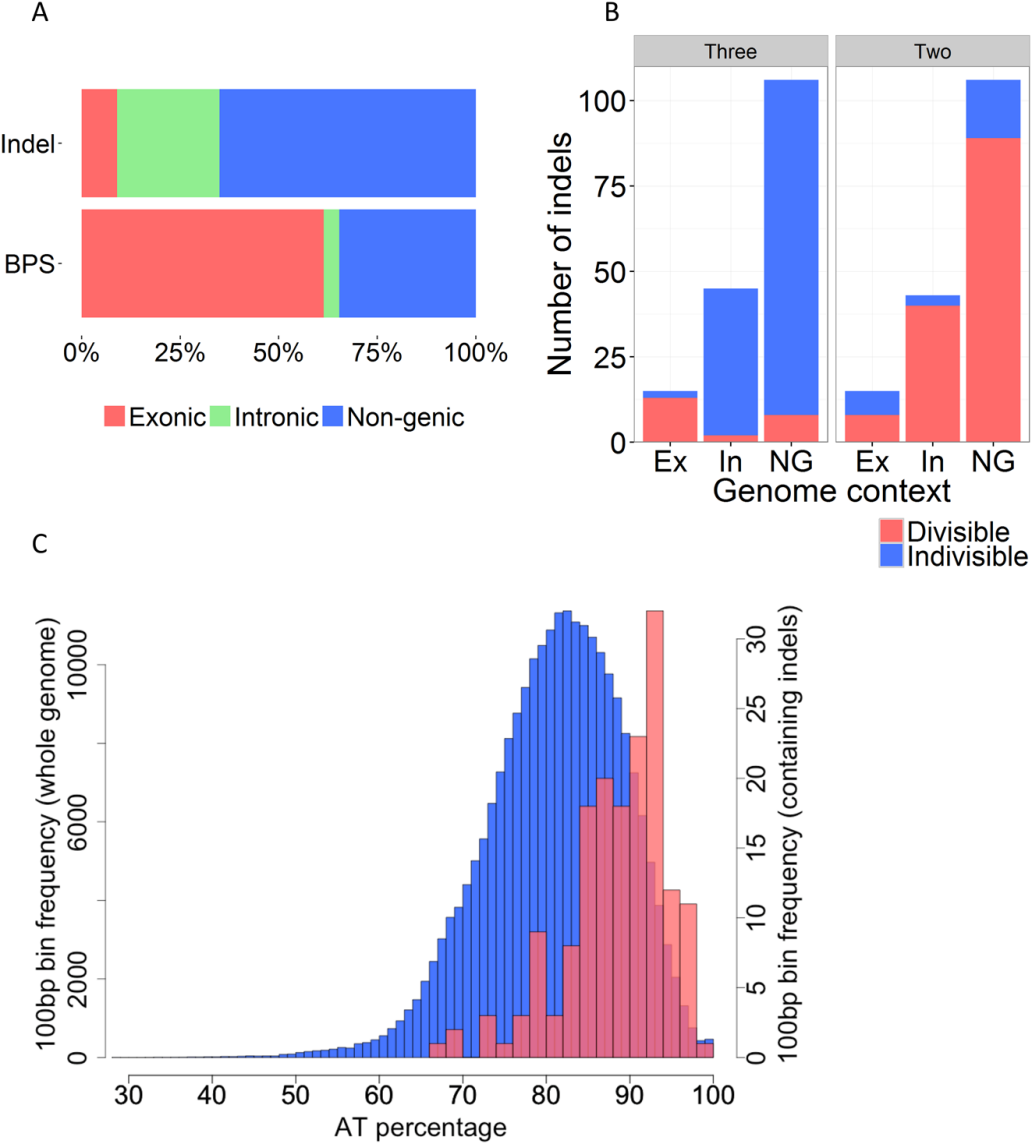
Indel distribution, size and AT content. (**A**) Relative distributions of indels and BPS in exons (Ex, red), introns (In, green) and non-genic loci (NG, blue). Indels (164 total from the 3D7 clone tree) were overrepresented in non-coding regions (non-genic and intronic), with only a small minority of indels (15/164, 9.1%) found in exons. In contrast, the distribution of BPS (78 pooled from all clone trees with known BPS coordinates) was similar to the underlying proportions of exonic, intronic, and non-genic sequence in the *P. falciparum* genome (raw data in Table 3). (**B**) Indel distribution in exonic, intronic, and non-genic loci, showing the number of indels whose nucleotide lengths are divisible (red) or indivisible (blue) by three (left) or two (right). Indels that are multiples of three preserve the reading frame when they occur in exons, and are overrepresented in exons compared with introns and non-genic loci (Table 4). In contrast, indels that are multiples of two bp are overrepresented from what is expected by chance in non-coding loci, reflecting the abundance of poly(AT) tracts in non-coding loci. (**C**) The 3D7 genome was divided into non-overlapping 100 bp bins, with the % AT content calculated for each bin and plotted as a histogram (blue bars, y-axis scale on left). The % AT content of the subset of all bins containing at least one indel was plotted separately (red bars, y-axis scale on right). The 100bp windows containing indels had a higher average AT content than those for the whole genome (88.1% and 80.6%, respectively) (*P* < 2.2 × 10^−16^, two-sample Welch *t*-test). Ex = Exon, In = Intron, NG = non-genic.

We analysed the genomic distribution and composition of *de novo* indels. Only 15/164 (9.1%) were observed in exons, a significantly different distribution from what would be expected by chance (*P* < 2.2 × 10^−16^, Pearson's Chi-squared test, Table 3). This contrasts with the distribution of BPS, which did not differ significantly from the background proportions of exons, introns and non-genic loci in the genome (Table 3, Figure 4A). 14/164 (8.5%) indels occurred outside of core genome coordinates (defined in (16)) close to chromosome ends. The AT content of combined inserted and deleted sequences was very high at 96.4% (83.7%, 99.3% and 99.4% in exons, introns and non-genic loci, respectively). This is high even by *P. falciparum* standards: in 3D7, the overall AT content is 76.3%, 86.5% and 86.4% in exons, introns and intergenic regions, respectively (2). Regardless of the coding or non-coding identity of the sequences, 100 bp regions containing an indel had a higher mean AT content (88.1%) than regions without identified indels (80.6%) (*P* < 2.2 × 10^−16^, Welch two-sample *t*-test, Figure 4C). All indels were flanked by at least one repeat of the same sequence. Collectively, these data show that indels occurred in highly AT-rich and repetitive sequences mainly in introns and outside of genes.

**Table 3.** Genomic distributions of indels and BPS

	3D7 clone tree		All wild-type clone trees	
	Observed indels (%)	Expected indels (%)	Observed BPS (%)	Expected BPS (%)
Exonic	15 (9.1)	86.3 (52.6)	48 (61.5)	41.1 (52.6)
Intronic	43 (26.2)	9.50 (5.79)	3 (3.8)	4.52 (5.79)
Non-genic	106 (64.6)	68.2 (41.6)	27 (34.6)	32.4 (41.6)
Total	164 (100)	164 (100)	78 (100)	78 (100)

The raw counts (and %) of observed indels from the 3D7 clone tree and BPS from all clone trees (3D7, HB3, Dd2, W2, KH1-01 and KH2-01) in exons, introns, and non-genic loci are shown, along with expected counts given a null hypothesis of the distribution being equal to the underlying genomic proportions (based on (2)). The distribution of indels between exons, introns and non-genic loci differed significantly from that expected by chance for indels (*P* < 2.2×10^−16^) but not for BPS (*P* = 0.511), Pearson's Chi-squared test.

Poly(AT) dinucleotide repeats are more common in non-coding DNA than in exons, while around 24% of *P. falciparum* proteins contain low-complexity amino acid homorepeats (60% asparagines and 23% lysines) (5,22). Of the latter, the most common codons are AAT (asparagine) and AAA (lysine) (4,22). We tested whether these sequence properties were reflected in the types of indel mutation arising in exonic and non-coding loci. 86.7% (13/15) of exonic indels were divisible by three bp, significantly more than expected by chance (*P* = 3.14 × 10^−5^, two-sided exact binomial test), while 87.2% (130/149) of indels outside of exons were multiples of 2 bp (*P* < 2.2 × 10^-16^, two-sided exact binomial test) (Figure 4B, Table 4). 12/15 (80%) of exonic indels involved asparagine or lysine residues and 7/15 (47%) caused expansion or contraction of poly(N) homorepeats (Supplementary Table S7). Thus, the underlying sequences inside and outside of exons are associated with distinct forms of indel mutation. This may be due to the processes of DNA polymerase slippage and unequal crossing over, combined with the tendency for purifying selection to purge frameshift-causing indels from the gene pool. These data support the observation that short tandem repeats (STR) across diverse genomes are highly polymorphic and prone to high mutation rates, with dinucleotide STR tending to have higher mutation rates than trinucleotide repeats (23).

**Table 4.** Breakdown of indels divisible by two and three nucleotides by genomic context

	Multiple of three bp		Multiple of two bp	
	% Observed indels (count)	*P*-value (expected ratio = one third)	% Observed indels (count)	*P*-value (expected ratio = one half)
Exon	86.7% (13/15)	3.14 × 10^−5^	53.3% (8/15)	1
Non-exon	6.71% (10/149)	9.61 × 10^−15^	87.2% (130/149)	<2.2 × 10^−16^

% (and raw count) of observed indels from the 3D7 clone tree which were divisible by two and three nucleotides are shown, broken down into those occurring inside and outside of exons (outside of exons includes both introns and non-genic loci). By chance, the expected proportions of indels divisible by three and two base pairs are one third and one half, respectively. *P*-values are derived from binomial exact tests. Indels that were multiples of three bp were more likely to occur in exons, while introns and non-genic loci were more likely to contain indels that were multiples of 2 bp. Data also shown in Figure 4.

### Mutation rate variation between isolates

It has been suggested that more drug resistant parasites have higher mutation rates, as this would increase the generation of novel genetic variants facilitating adaptive evolution (24–26). The KH2-01 parasite was artesunate slow-clearing *in vivo* and possessed several of the highly differentiated SNPs in DNA repair genes identified by Miotto *et al.* (11), including P203S in *mlh1*, S506I in *pms1*, and C2010S in *rad50*, whereas KH1-01 was artesunate fast-clearing and wild-type at these loci. The pooled mutation rate from all six *P. falciparum* isolates was 2.45 × 10^−10^ (95%CI, 1.70 × 10^−10^–3.20 × 10^−10^) BPS/ELC/bp, consistent with other estimates (6,7). The rates did not differ significantly between any isolate pairing (Table 1), including KH2-01 v KH1-01 (two-sample Welch *t*-test). Thus, we found no evidence of a BPS hypermutator phenotype in either the artesunate fast-clearing or slow-clearing Cambodian field isolates.

**Figure 5.**
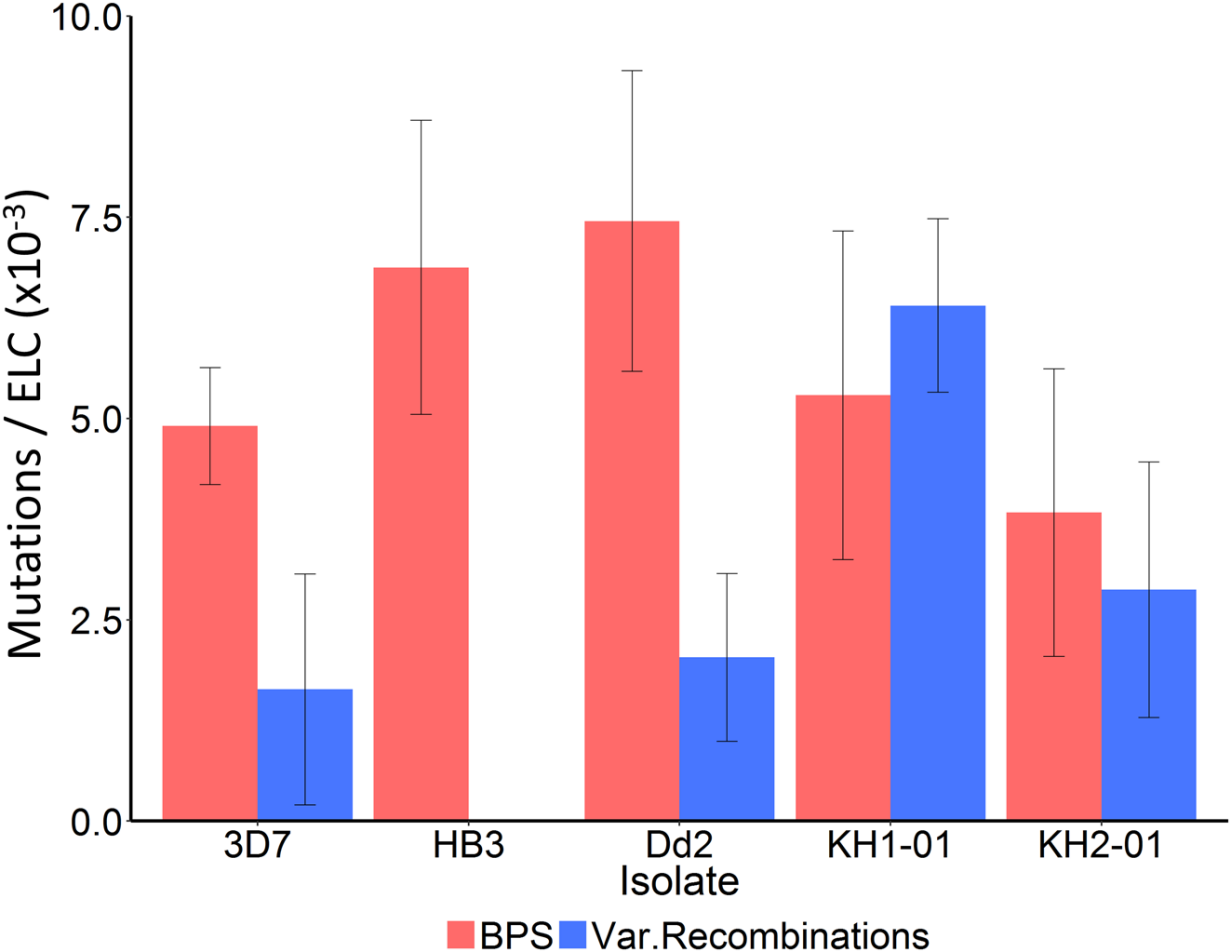
Base pair substitution (BPS, red) and *var* exon 1 recombination rates (blue) shown per erythrocytic life cycle (ELC) in all isolates. BPS rates did not differ significantly between the different isolates, nor between the pooled long-term culture-adapted laboratory isolates (3D7, HB3 and Dd2) and the combined Cambodian isolates (KH1-01 and KH2-01) (all pairwise comparisons by two-sample Welch *t*-test). In contrast, the combined *var* gene exon 1 recombination rate in the Cambodian field isolates was significantly higher than for the combined laboratory isolates (*P* = 0.0306, two-sample Welch *t*-test after Bonferroni correction for multiple comparisons). The KH1-01 *var* exon 1 recombination rate was 6.40 × 10^−3^ (95%CI 4.30 × 10^−3^–8.51 × 10^-3^) recombining *var* pairs per ELC, or one parasite with recombined *var* genes for every 156 (95%CI 117–233) parasites following a single 48-h ELC. Error bars show standard error of the mean.

Unlike BPS, rates of non-allelic homologous recombination (NAHR) in and around *var* genes differ significantly between long-term laboratory adapted isolates (6). NAHR produces *var* gene sequence mosaicism during erythrocytic division, potentially adding to PfEMP1 antigenic diversity within infected people. We analysed the rate of *var* gene NAHR in our two Cambodian isolates, which are more biologically relevant as they are recently derived from their natural *in vivo* environment. We identified 27 pairs of recombining *var* genes in these isolates (Supplementary Table S8). Unlike BPS, the *var* gene exon 1 recombination rates were significantly higher in the Cambodian isolates than in the long-term culture-adapted laboratory isolates (*P* = 0.0306, two-sample Welch *t*-test with Bonferroni correction for multiple comparisons (18)) (Figure 5 and Supplementary Table S9). In KH1-01, there would be approximately one *var* gene recombination event, potentially producing an antigenically novel structure, for every 156 parasites (95%CI 117–233) per 48-h ELC. That is 1.21-fold higher than the BPS mutation rate in KH1-01 (Supplementary Table S9).

We investigated whether the higher *var* exon 1 recombination rate in the Cambodian isolates correlated with greater *var* gene transcriptional activity. *Var* genes are divided into three main families, A, B or C, based on phylogenetic analysis (27). Both the variety and quantity of *var* gene expression in wild parasites drop when they enter *in vitro* culture, with group B and C *var* genes predominating (28–30). Sampled Dd2 subclones from our clone tree expressed a very limited range of their *var* gene repertoire, with just two *var* genes dominating expression (Supplementary Figures S5 and S6). In contrast, no single *var* gene dominated consistently out of the five KH1-01 subclones analysed, and there were at least 37 *var* genes transcribed in the pooled KH1-01 dataset (Supplementary Figures S5 and S6). Thus, the more recently culture-adapted KH1-01 field isolate had both a higher *var* recombination rate and a greater variety of expressed *var* genes than the long-term culture-adapted Dd2 isolate. However, the *var* genes that underwent recombination were not those dominantly expressed in the preceding clone tree generation (Supplementary Figure S5), so a direct relationship between expression and recombination of specific *var* genes was not observed.

## DISCUSSION

We have used mutation accumulation lines to study how spontaneous mutation across the *P. falciparum* genome sculpts the parasite's unusual genomic architecture. We found evidence of significant transition bias driven by an overrepresentation of G:C→A:T substitutions, expected to push the genome to its current skewed base pair composition of ∼80% AT. This is consistent with population genetic data suggesting an underlying mutation bias that favours G/C→A/T (31). This mutational bias may contribute to producing AT-rich, low-complexity sequences, particularly in non-coding loci where purifying selection is generally relaxed. Such AT-rich repetitive sequences can then cause DNA polymerase slippages and unequal crossing over events, as tandem repeats are prone to slipped-strand mispairing during DNA replication (32,33), and are associated with indel mutations (34). While previous studies have identified high indel variation between *P. falciparum* genomes (16,35), we have demonstrated the prolific rate at which these highly dynamic features occur during mitotic division: about 10-fold more frequently than *de novo* BPS. This is unusual amongst eukaryotes; in most other organisms studied BPS outnumber indel mutations (36–38), with substitution to indel ratios in sampled primate and bacterial genomes of 9.89 and 19.61, respectively (38). These data pose fundamental questions about *P. falciparum* genome biology. What is the evolutionary history of skewed genome composition in the *Plasmodium* family tree? What are the molecular mechanisms driving high rates of G:C→A:T transition? And what are the biological consequences of high AT content with high indel mutation rates; do they serve any adaptive roles for the parasite?

*P. falciparum* is not unique among *Plasmodium* species in possessing an AT-rich genome (Supplementary Table S1 and Figure 1). Closely related parasites in the *Laverania* subgenus from which *P. falciparum* emerged (39), such as *P. reichenowi* (40) and *P. gaboni* (41), also have markedly skewed genome compositions (80.7% and 79.4% AT, respectively). This suggests that systematic substitution bias driving high AT content predates the divergence of the *Laverania* group. How far back in evolutionary time does genome composition bias extend? The related primate parasites *P. cynomolgi, P. knowlsei* and *P. vivax* (42) have more even base pair compositions (59.4%, 60.8% and 59.4% AT, respectively), though this may be due to recent evolution towards higher GC, away from an AT-rich common *Plasmodium* ancestor (3). The bird parasite and evolutionary outgroup to *Plasmodium, Haemoproteus tartakovskyi*, also parasitizes erythrocytes and has high AT content, at 75% (43). The Apicomplexan parasites *Babesia bovis, Theileria parva* and *Toxoplasma gondii*, all outside of the *Plasmodium/ Haemoproteus* group (44), have more even AT contents (58.2%, 65.9% and 47.7%, respectively) (Figure 1) (45). Thus, a degree of AT bias may have evolved at least as far back as the common ancestor of *Plasmodium* and *Haemoproteus*. This model can be tested as more parasite genomes become available and phylogenetic trees with genome-wide AT contents (as in Figure 1) are more comprehensive.

Overrepresentation of G:C→A:T transitions and a mutation bias in favour of AT-richness is seen in diverse organisms (46–52), and transition mutations outnumber transversions in most organisms with intact DNA repair machinery (53–59). A major reason for transition bias and elevated G:C→A:T mutation rates is cytosine deamination to uracil, which is a common spontaneous biochemical change in DNA (60). We did not find a statistically significant enrichment of CpG or TpC dinucleotides at *de novo* C→T transitions, which would be expected if 5-methylcytosine deamination to thymine were playing a role. However, this finding should be cautioned by the low number of *de novo* C→T mutations identified.

In bacteria, obligate intracellular symbionts tend to evolve high AT-content (61,62) as guanine and cytosine nucleotides require higher energy for biosynthesis and are less readily available intracellularly. Relaxed selection pressure in intracellular parasites causes gene loss (63), and may facilitate the natural tendency for mutation to drive the genome towards high AT. Alterations in DNA repair and polymerase pathways also affect base pair composition (64–68), and DNA repair gene mutations or pathway degradation are often seen in obligate intracellular pathogens (61). Several features of *P. falciparum* DNA repair are unusual, such as the absence of a canonical non-homologous end joining pathway (69,70). The parasitized intraerythrocytic environment is also rich in reactive oxygen species, reactive nitrogen species, and NO radicals (71–74), which can cause cytidine deamination to uracil, driving C→T transitions (60,75). It may be that a combination of factors are responsible for *P. falciparum* G:C→A:T substitution bias, including cytosine deamination, exacerbated by exposure to intraerythrocytic oxidising agents, combined with underlying abnormalities in DNA repair. Further research is necessary to investigate these influences, for example, by culturing *P. falciparum* in oxidising conditions or generating lines with a range of genetically modified DNA repair activity and monitoring the effect on BPS rates and bias.

There are at least three potential biological consequences of widespread AT-rich, repetitive loci associated with high indel mutation: expansion and contraction of amino acid homorepeats, alterations to gene expression through indels in AT-rich regulatory DNA, and increased rates of copy number variation (CNV). Most of the exonic indels we observed contained asparagine and/or lysine residues in repetitive sequences, suggesting these low-complexity regions undergo a high rate of indel mutation. Various adaptive roles for these repeats in *P. falciparum* proteins have been suggested (76), such as tRNA sponges (77) and antigenic variation for immune evasion (78,79). They may provide a general platform for evolutionary adaptation, with the potential for mutation and selection to drive neofunctionalisation of the asparagine repeats in different contexts (76). AT-rich non-coding DNA surrounding genes is also likely to undergo a high indel rate. This could affect gene expression, as untranslated regions up- and down-stream of genes contain both enhancer and repressor elements (reviewed in (80)). Lastly, CNVs are correlated with repetitive sequences such as *Alu* repeats in human and mouse genomes, likely due to NAHR processes (81–84). *P. falciparum* CNVs contribute to the evolution of antimalarial drug resistance, for example amplification of *pfmdr1* and *gch1* genes driving resistance to mefloquine and antifolates, respectively (85,86). The amplicon breakpoints in *pfmdr1* and *gch1* occur in microsatellites and monomeric A/T tracts (87,88). It has been estimated that there are 37 177 monomeric A/T tracts in the *P. falciparum* genome, providing ample scope for the *de novo* emergence of CNV through NAHR-like processes (88). The mechanism first involves random duplication of DNA between two A/T tracts, followed by a more precise amplification of the sequence under selection (89). Thus, G:C→A:T transition bias may help generate widespread repetitive AT-rich low-complexity regions, both inside and outside of coding sequences, ultimately facilitating a form of microstructural genomic dynamism providing additional fuel for adaptive evolution.

We found no significant differences in BPS mutation rates between isolates, regardless of their geographic origins or the degree of drug resistance. This is consistent with a recent population genetic analysis which found no evidence of hypermutation in Southeast Asian parasites, including those possessing multiple highly differentiated SNPs in DNA repair genes found in the KH2 sub-population (90); though a mild mutator phenotype has been reported in a yeast model of the *mlh1* 203S variant (91). A more systematic evaluation of multiple isolates is needed to formally demonstrate that artemisinin resistant KH2 parasites, on average, have no difference in mutation rates to artemisinin sensitive parasites. In contrast, rates of *var* gene NAHR differed significantly between isolates, with the two Cambodian lines having higher rates than the long-term culture-adapted laboratory isolates. The Cambodian KH1-01 parasite also expressed a greater variety of its *var* gene repertoire than the Dd2 isolate. This is expected, as the Cambodian isolates were more recently taken from the *in vivo* setting where *var* gene transcriptional activity is higher (28–30). If transcription somehow opens up the chromosome to facilitate recombination, then higher levels of a more diversely expressed *var* gene repertoire could contribute to the higher recombination rates observed in KH1-01 and KH2-01. This could also explain why group A *var* genes have a lower recombination rate than group B or C *var* genes in the clone trees (discussed in Claessens *et al.* 2014 (6)) – group A *var* recombination may be more common *in vivo* where they are expressed more often, particularly in severe malaria (92,93). However, correlation does not demonstrate causation; further research is needed to investigate any mechanistic relationship between *var* gene transcription and mitotic recombination. *Var* gene recombination patterns *in vivo* are currently unexplored.

Even a relatively low parasitaemia of 0.01% (∼500 parasites/μl of blood) represents ∼1 billion parasites circulating in a single symptomatic human infection. Every two days, one would expect ∼6 million substitutions, 55 million indels and 4 million newly created mosaic *var* exon 1 sequences to emerge through *de novo* mutation inside an infected person. Thus, a continuous wellspring of genetic variation, particularly small indels, is generated across the ∼200 million symptomatic malaria cases each year. Effective population size, more relevant for adaptive evolution than total population, has been estimated at around 10^4^–10^6^ parasites in *P. falciparum* (31,94,95, http://dx.doi.org/10.1101/056291). These numbers highlight the need for effective artemisinin partner drug combinations (96), optimal patient adherence and high medicine quality to constrain resistance evolution. The C580Y mutation in *kelch13* implicated in artemisinin resistance (97–100), for example, has arisen multiple times in Southeast Asia (100), and *kelch13* mutations are detectable at low frequency in Africa (101). The great challenge facing malaria control efforts is to keep ahead of the curve, and eliminate these parasites before the evolutionary process has allowed particularly dangerous combinations of mutations, with high levels of resistance combined with strong biological fitness, to emerge and spread through parasite populations.

## ACCESSION NUMBERS

Whole genome sequences can be found in the European Nucleotide Archive with accession numbers available in Supplementary Table S2.

## Supplementary Material

Supplementary Data
