# Supplementary material for: Extreme mutation bias and high AT content in Plasmodium falciparum
Source: Nucleic Acids Res. 2016 Dec 19;45(4):1889–901. doi: 10.1093/nar/gkw1259 (PMC5389722; doi:10.1093/nar/gkw1259)
Supplement: Supplementary Data [file gkw1259_Supplementary_Data.zip › Supplementry_Figures.pdf]

Figure S1. Generating *P. falciparum in vitro* clone trees

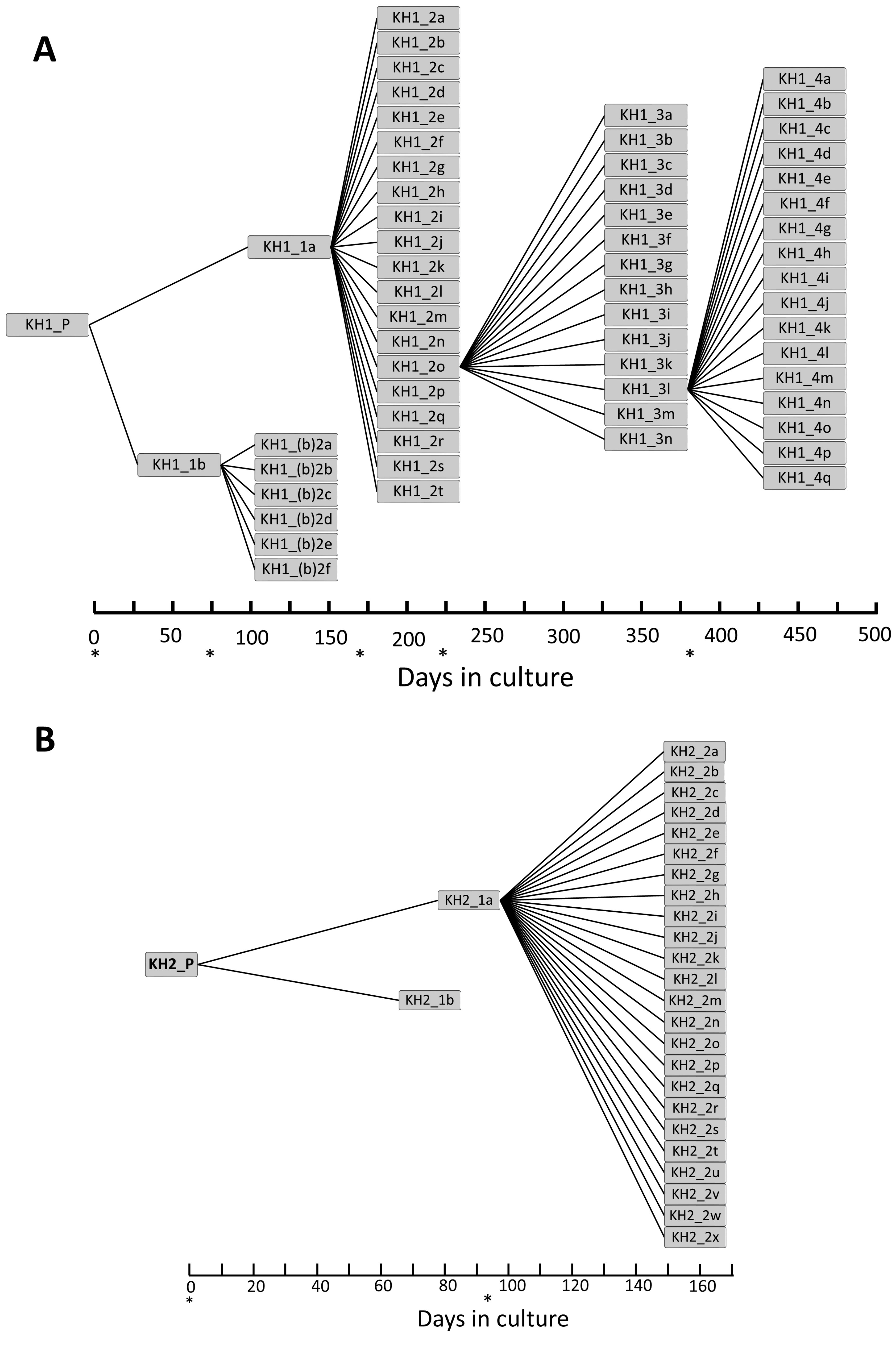

Clone trees for KH1-01 (**A**) and KH2-01 (**B**) are shown. Each box indicates a whole-genome sequenced clone, and branch points indicate a round of limiting dilution cloning. Subclones were chosen for the next round of limiting dilution at random, forming the branching trees. Asterisks on the x-axes indicate when clonal dilutions were performed. Clone trees for the long-term-adapted laboratory isolates 3D7, HB3, Dd2, and W2 are shown in Claessens *et al*. 2014 (ref 6).

Figure S2. Correlation between clone tree variables and *de novo* BPS

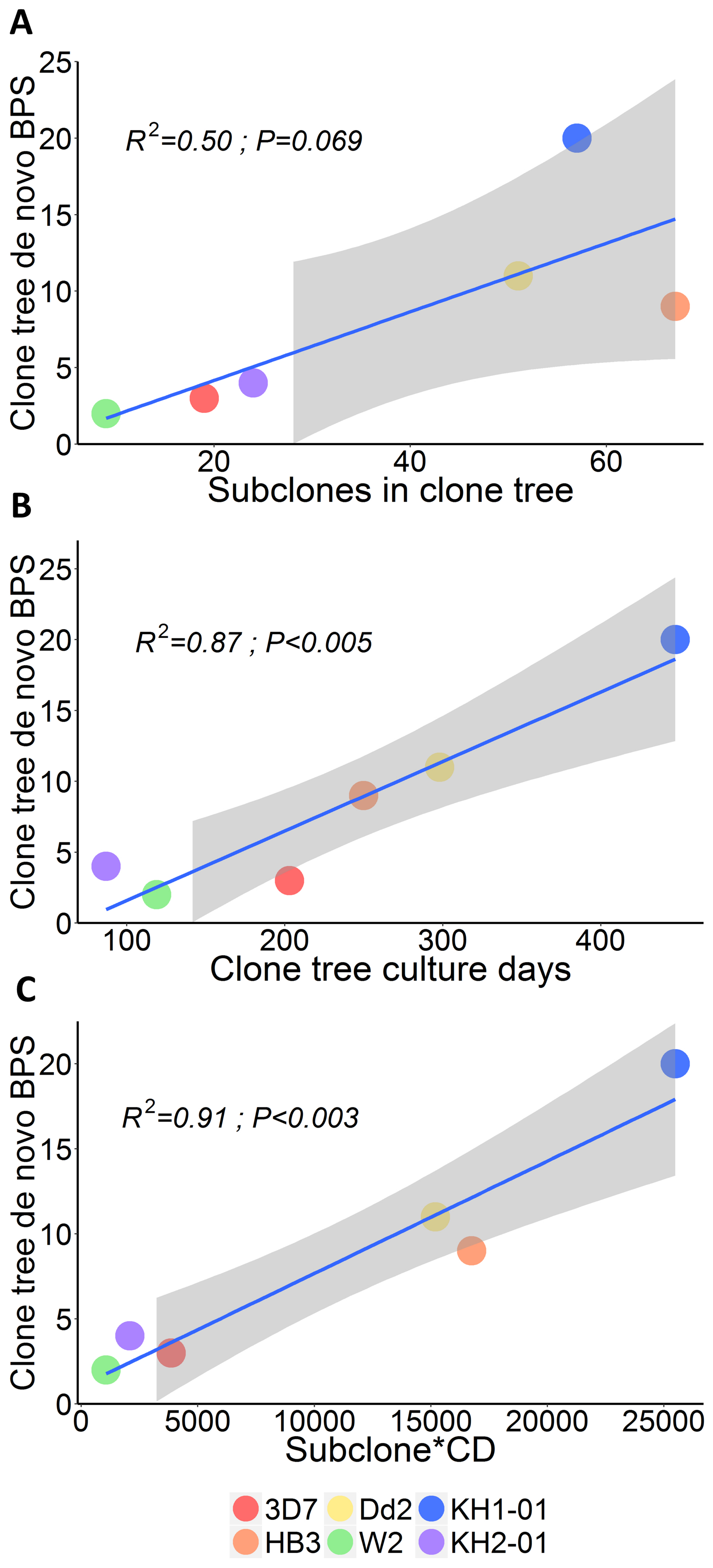

Plots show linear regression models for the relationships between the number of *de novo* BPS identified in each clone tree and three clone tree variables: the number of subclone genomes analysed (**A**), the total days in culture (**B**), and the multiplication of the number of subclone genomes analysed and the total days in culture, referred to as Subclone*CD (**C**). Each point is from one of the six clone trees: 3D7, HB3, Dd2, W2, KH1-01 and KH2-01. All three clone tree variables show a positive correlation with *de novo* BPS: **A,** r^2=0.505, *P*=0.0691; **B,** r^2=0.865, *P*=0.00451; and **C,** r^2=0.908, *P*=0.00211. The strongest relationship was for Subclone*CD, reflecting both the number of subclone genomes analysed and the total time in culture for each clone tree. This is expected given that mutation is a stochastic process and so more *de novo* mutations will be identified from larger clone trees (i.e. those with more subclones and greater culture times). In all cases, first-generation subclones, for which the preceding culture time is unknown, were excluded for both *de novo* BPS and total subclones analysed. This is because only BPS in subclones for which culture time is known can be used to calculate mutation rates.

Figure S3. Visualisation of 3D7 indel hits in Illumina sequence data

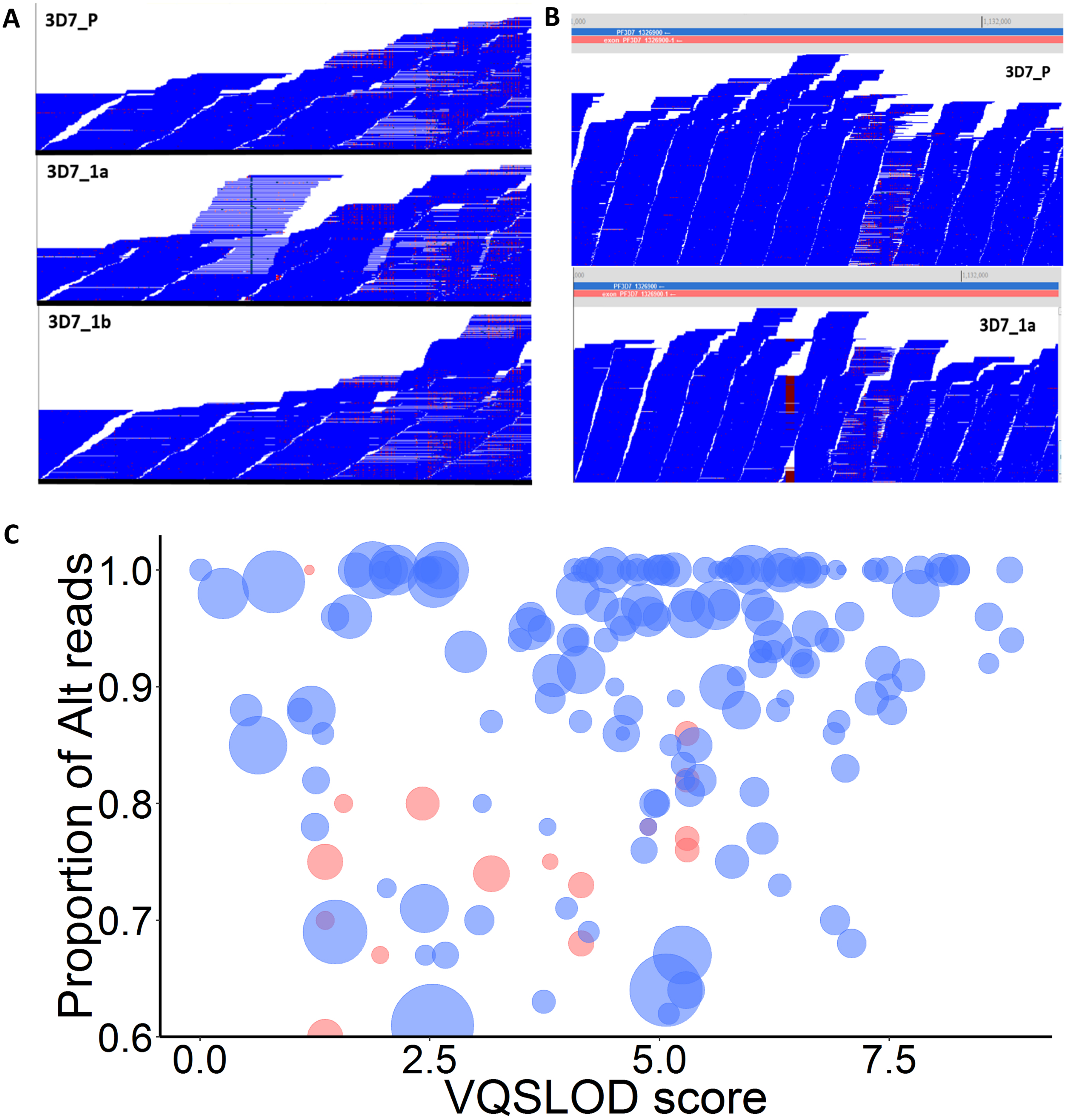

Insertion (**A**) and deletion (**B**) in subclone 3D7_1a, visualised in LookSeq (ref 14). **A:** The insertion is visualised by the extra nucleotide sequence from the reads aligning in this region, which LookSeq adds beneath each read thus creating a "spaced out" appearance to the read stack in 3D7_1a compared with its parent 3D7_P or other 3D7 subclones such as 3D7_1b. **B:** The deletion in subclone 3D7_1a is shown by the dark red section of nucleotides in the read stack, which are absent from the read sequences but present in the 3D7 reference genome against which the reads are aligned. **C:** VQSLOD score (from GATK's VQSR, x-axis) and the proportion of reads indicating an indel (y-axis) from all 180 3D7 indel hits. If the read pileup visualised in LookSeq did not unequivocally show an indel in a progeny sample and its absence in its parent, it was labelled "false" (n=16, coloured red). "True" indels (n=164) are coloured blue. The diameter of each bubble is proportional to the total high-quality read depth mapping to that locus (median: 22x). VQSLOD is the log odds ratio of the hit being a true variant versus being a false variant under the trained Gaussian mixture model. As expected, hits with a higher VQSLOD score, greater proportion of reads indicating an indel and higher overall coverage were more likely to be "true".

Figure S4. BPS spectrum in progeny of three experimental *P. falciparum* genetic crosses

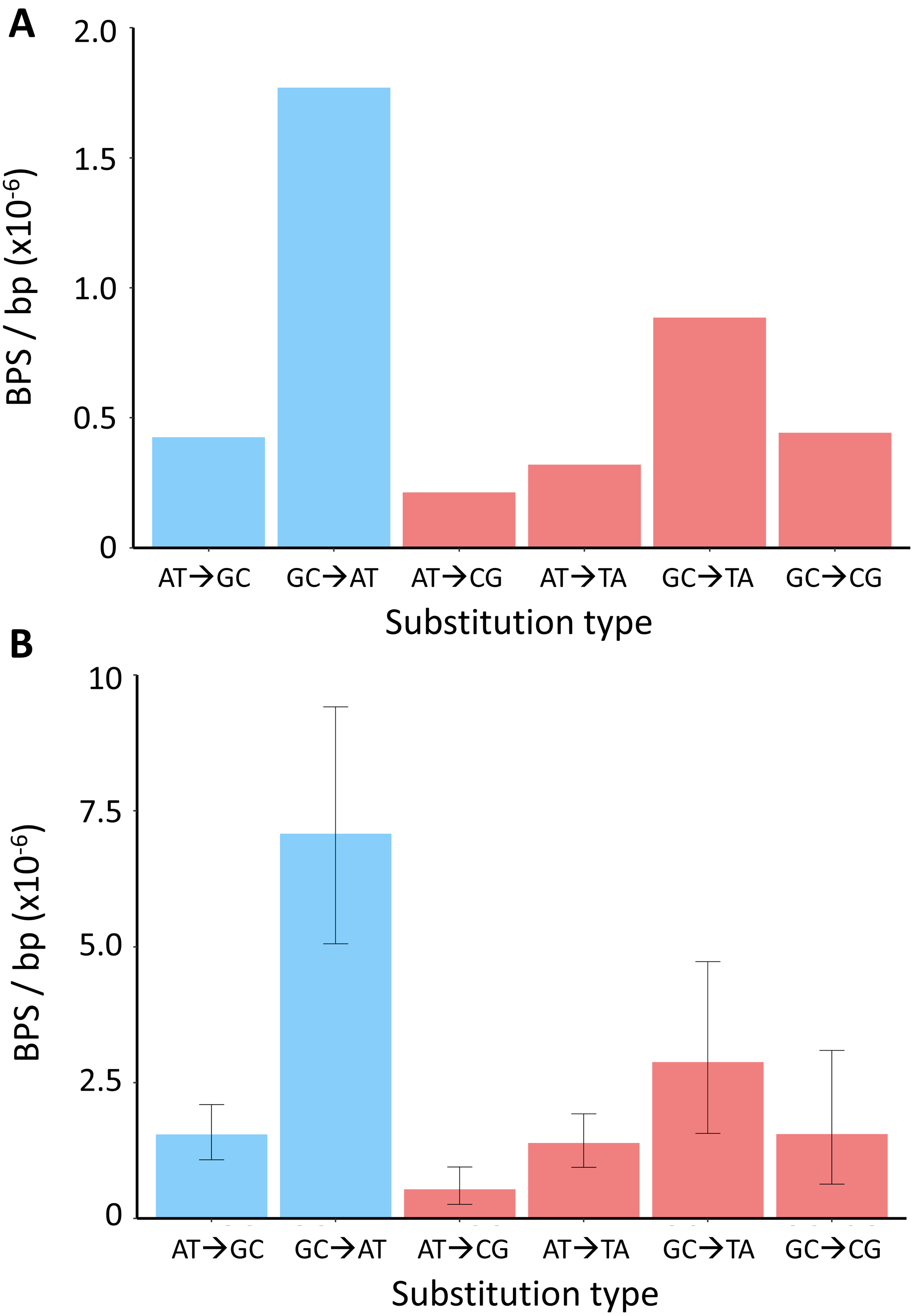

32 BPS were identified in the progeny of three experimental genetic crosses: HB3xDd2, HB3x3D7, and 7G8xGB4 (BPS details in Table S5). The raw counts for each BPS type are shown in Table 2. **A:** Dividing raw BPS counts from the crosses dataset for each substitution type by the total number of A/T (18779800) or G/C (4520200) nucleobases in the *P. falciparum* genome yields the BPS/bp values plotted here. The pattern is similar to that observed in the clone trees (Figure 3), with G:C→A:T substitutions occurring at the highest rate per bp. **B:** Combining the clone tree and crosses data gives a combined dataset of 117 *de novo* BPS, with BPS/bp values shown here (same derivation as in A). The distribution of BPS between substitution types deviated significantly from what would be expected if all substitutions occurred at equal rate per nucleotide ($\chi^2 = 27.52$, $df = 5$, $P = 4.517 \times 10^{-5}$, Pearson's Chi-squared test). Bars show Clopper-Pearson (exact) 95% confidence intervals. Transitions are in blue and transversions in red.

Figure S5. *Var* gene expression in selected subclones from the Dd2 and KH1-01 clone trees

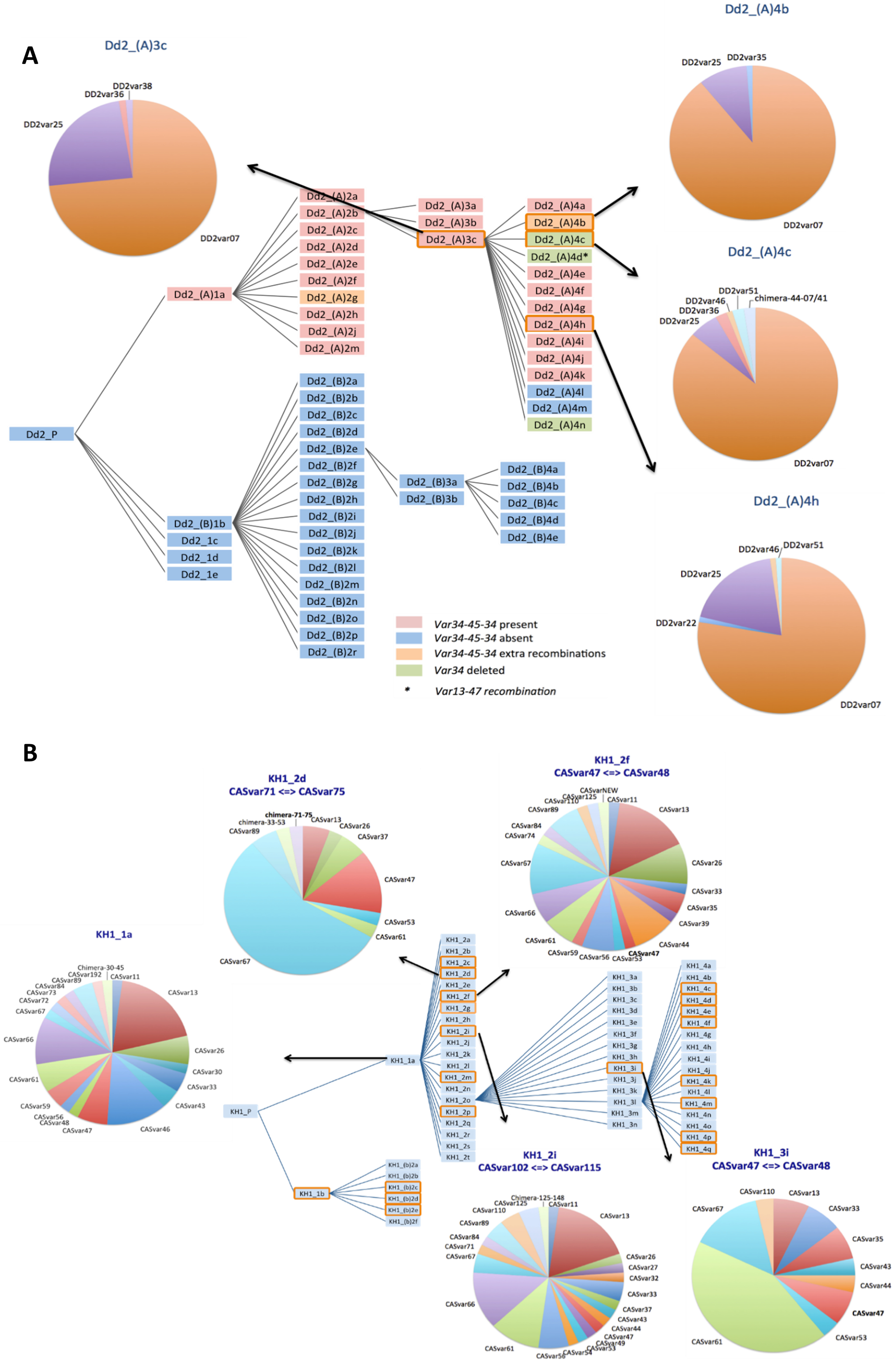

cDNA derived from synchronized trophozoite-stage Dd2 and KH1-01 subclones was used as template to amplify *var* gene DBLα regions using universal primers. Proportions of different *var* gene transcripts in the selected subclones are represented in pie charts. **A:** The Dd2 clone tree. Frequent recombination between *Dd2var34* and *Dd2var45* was observed, indicated by subclone rectangles of different colours. However, transcripts for *Dd2var34* or *Dd2var45* were not detected in any of the selected subclones, including the parent subclone to that clone tree generation (Dd2_(A)3c). Conversely, *Dd2var07* and *Dd2var25* were dominantly expressed in all tested subclones, but these *var* genes were not involved in recombination events. **B:** KH1-01 clone tree. Orange rectangles indicate subclones in which a *var* gene recombination event was detected. Five subclones were selected for *var* gene RT-PCR; the two recombining *var* gene names are written under the subclone name. For example, in subclone KH1_2d, a chimeric *var* gene resulting from the recombination between *CASvar71* and *CASvar75* was detected (this chimera represented 3% of all transcripts in KH1_2d). These data show that all Dd2 subclones had the same two dominant *var* genes expressed, *Dd2var07* and *Dd2var25*. In contrast, the same *var* gene is not dominantly expressed in any two of the selected Cambodian isolates, and several isolates lack a clear dominant *var* gene (e.g. KH1_1a, KH1_2f, and KH1_2i).

Figure S6. Overall *var* gene expression profile in Dd2 (A) and KH1-01 (B) clone trees

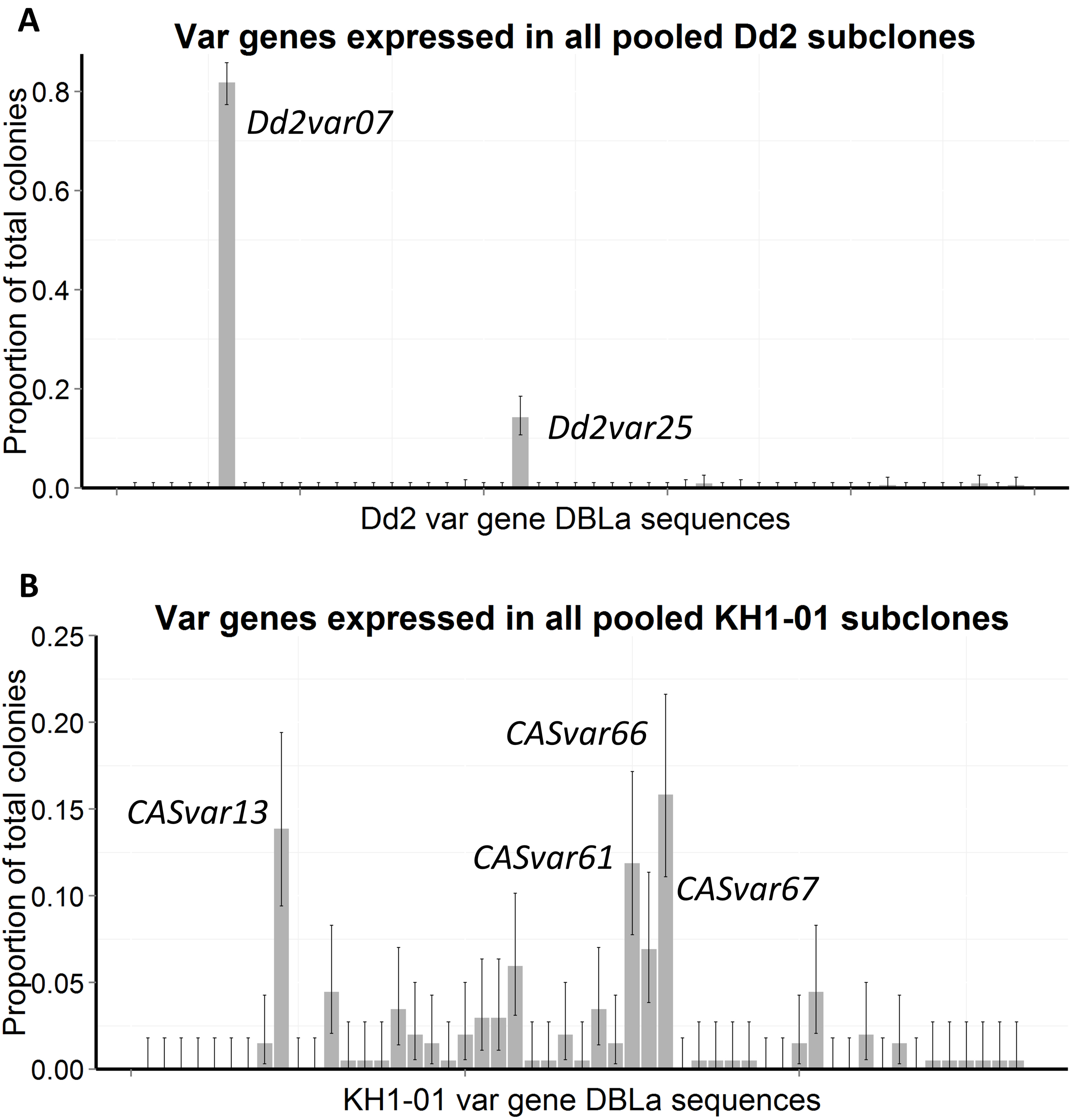

Plots show the proportion of pooled DBLα transcript sequences taken up by each *var* gene sequence in that isolate. *Var* genes representing the highest proportions of expressed sequences are labeled. Just two *var* genes, *Dd2var07* and *Dd2var25*, dominate expression in the Dd2 clone tree, with very little expression in all others. In contrast, there were more *var* genes found in >5% of pooled sequences in KH1-01, and no single *var* gene dominated expression. This is consistent with more rapid *var* gene switching and a greater variety of expressed *var* genes in KH1-01 parasites compared with Dd2. Error bars show Clopper-Pearson (exact) 95% confidence intervals.
