# Supplementary material for: Extreme mutation bias and high AT content in Plasmodium falciparum
Source: Nucleic Acids Res. 2016 Dec 19;45(4):1889–901. doi: 10.1093/nar/gkw1259 (PMC5389722; doi:10.1093/nar/gkw1259)
Supplement: Supplementary Data [file gkw1259_Supplementary_Data.zip › Supplementary_Appendix.docx]

Appendix: Supplementary Notes

# S1. False positives and false negatives in the clone trees

Mutation rate analyses may suffer from two problems: false positives, mutations that are counted but not actually genuine; and false negatives, mutations that are not counted but are genuine. We addressed false positives by using validated SNP calling methods for *P. falciparum* Illumina data, manually inspecting all putative mutations on LookSeq, and performing capillary sequencing to validate a subset of BPS, *var* gene recombinations, and indels (see (1) and Methods).

Because genome accessibility differs between the isolates studied, the false negative rate may also differ, and this must be taken into account when comparing BPS mutation rates between isolates. Specifically, 3D7 has the most accessible genome because its reference sequence is the most complete. We mapped the HB3 and Dd2 reads to both the 3D7 reference sequence and to versions of the HB3 and Dd2 reference sequences (neither of which are as complete as the 3D7 reference sequence) (1). KH1-01 and KH2-01 are wild isolates with no reference sequences available, and so were mapped only to the 3D7 version 3 genome. It is therefore likely that we missed more mutations in KH1-01 and KH2-01 than in 3D7, HB3, or Dd2. This could explain why the BPS mutation rates in KH1‑01 and KH2-01 were slightly lower than for the laboratory isolates (mean 2.90x10^-10^ BPS/ELC/bp for laboratory isolates (3D7, HB3, and Dd2), compared with 2.27x10^-10^ and 1.64x10^-10^ BPS/ELC/bp for KH1‑01 and KH2-01, respectively, though the differences were not statistically significant).

This assumption can be formally tested: the median percentage of the 3D7 reference genome covered by ≥5x and ≥10x reads for all clone trees is shown in Table S1.1. As expected, 3D7 samples have the highest median percentage of the 3D7 genome covered (99.4% covered by ≥5 reads). Median % 3D7 genome with ≥5x coverage was still >94% for both KH1-01 and KH2-01, and no individual isolate from any clone tree had <80% of the genome covered with ≥5 reads. A breakdown of coverage across the 3D7 version 3 reference genome for every isolate is shown in Table S2.

| **Isolate** | **Median % 3D7 genome with ≥5x coverage (IQR)** | ***P*-value for comparison with 3D7: ≥5x coverage** | **Median % 3D7 genome with ≥10x coverage (IQR)** | ***P*-value for comparison with 3D7: ≥10x coverage** |
| --- | --- | --- | --- | --- |
| 3D7 | 99.4 (2.5) | - | 97.1 (9.4) | - |
| HB3 | 96.7 (1.9) | 2.16x10^-8^ | 95.6 (5.8) | 0.00157 |
| Dd2 | 96.4 (1.3) | 4.13x10^-8^ | 94.8 (4.7) | 0.00259 |
| W2 | 96.4 (0.9) | 4.84x10^-5^ | 94.7 (4.0) | 0.0228 |
| KH1-01 | 95.6 (4.7) | 8.96x10^-9^ | 89.6 (10.2) | 5.65x10^-6^ |
| KH2-01 | 94.7 (2.1) | 1.94x10^-6^ | 87.2 (5.7) | 7.48x10^-5^ |

**Table S1.1. Median % 3D7 version 3 reference genome with ≥5x and ≥10x coverage.** IQR = interquartile range. *P*-value columns show *P*-values from Wilcoxon rank sum tests comparing coverage with >5x and >10x reads in each isolate vs the 3D7 isolate.

One method of assessing how many mutations are being missed in KH1-01 and KH2-01 compared with the laboratory isolates is to see how many mutations in HB3 and Dd2 were found exclusively in the HB3 and Dd2 reference sequences and not in the 3D7 reference sequence. 9 of 64 (14%) BPS in the Dd2, W2, and HB3 clone trees were found in the Dd2 or HB3 reference genomes but not in the 3D7 version 3 reference (Table S1.2), suggesting that we underestimated the BPS mutation rates in KH1‑01 and KH2-01.

| **Isolate** | **Total BPS** | **BPS found only in the non-3D7 reference genome (%)** | **Non-3D7 reference genome** |
| --- | --- | --- | --- |
| Dd2 | 18 | 5 (28%) | Dd2 |
| W2 | 11 | 1 (9%) | Dd2 |
| HB3 | 17 | 1 (6%) | HB3 |
| Total | 46 | 7 (15%) |  |

**Table S1.2. Proportion of BPS that are found only in the non-3D7 reference genomes.**

An explanation for the higher incidence of BPS called only in the non-3D7 reference genomes, despite high overall coverage across the 3D7 reference, is that the missing BPS occur in regions outside of the “core genome,” which are difficult to map and call variants in. If BPS were not adequately called in KH1-01 and KH2-01 genomes outside of the core 3D7 sequence, one predicts that a higher proportion of KH1-01 and KH2-01 BPS would occur in the core sequence of 3D7 than in the core sequences of other isolates. We tested this hypothesis by defining a core 3D7 nuclear genome based on coordinates for the 3D7 version 3 reference sequence provided by Alistair Miles (2). The core genome excludes the start and end of each chromosome (removing repetitive sequences in the telomeres and highly polymorphic genes in the subtelomeres), and the internal regions of chromosomes 4, 7, 8, and 12 that contain *var* gene clusters. The 85 BPS across all six clone trees were divided into those that occurred within or outside of this core genome (BPS that only mapped to the HB3 or Dd2 reference sequence were defined as outside of the core). For laboratory isolates (3D7, HB3, Dd2, and W2), 46/61 (75.4%) of BPS occurred within the core 3D7 nuclear genome (Table S1.3). This compares with 23/24 (95.8%) of BPS from the KH1-01 and KH2-01 isolates being found within the core 3D7 genome. If the total pool of substitutions (n=61) from laboratory isolates had the same distribution within core and non-core genomes as the Cambodian field isolates (96%), then one expects ~59/61 BPS to be within the core genome. This differs significantly from what was observed (*P*<0.002, Fisher’s exact test).

This supports the view that some BPS in KH1-01 and KH2-01 were missed because they were situated outside of the core 3D7 genome in regions that are difficult to call, for example, subtelomeres and polymorphic antigenic loci such as *var* genes. In conclusion, the false negative rate for calling BPS in KH1-01 and KH2‑01 is likely to be higher than that in the laboratory isolates due to the absence of reference genomes, and BPS outside of the 3D7 core genome being missed. This will result in an underestimation of the BPS mutation rate in KH1-01 and KH2-01 compared with the laboratory isolates. However, this effect would be expected to affect KH1-01 and KH2-01 to a similar degree.

| **Isolate** | **3D7 core (%)** | **Not core** | **Total** |
| --- | --- | --- | --- |
| 3D7 | 10 (66.7) | 5 | 15 |
| HB3 | 16 (94.1) | 1 | 17 |
| Dd2 | 10 (55.6) | 8 | 18 |
| W2 | 10 (90.9) | 1 | 11 |
| KH1-01 | 19 (95.0) | 1 | 20 |
| KH2-01 | 4 (100) | 0 | 4 |
| Total | 69 (81.2) | 16 | 85 |

**Table S1.3. Proportion of BPS occurring within and outside of the core 3D7 nuclear genome.** Aggregating the mutations for all laboratory isolates yields 61 BPS, of which 46 (75.4%) occurred within the core 3D7 nuclear genome. In contrast, 23/24 (95.8%) of BPS from the KH1-01 and KH2-01 Cambodian clone trees combined were found within the core 3D7 nuclear genome.

Finally, there are evolutionary forces inherent to the clone tree process that result in mutations being missed, though these should be similar between different clone trees. First, even in the absence of selection pressures such as antimalarials and human immunity, some mutations will be deleterious and removed from the population by purifying selection, e.g. if they disrupt red blood cell invasion or intraerythrocytic development. This could explain why a significant proportion of indels in coding regions were divisible by three, such that the protein’s reading frame was maintained (discussed in the main text). Second, most neutral *de novo* mutations are quickly lost from populations after they arise simply due to random genetic drift. The frequency of a new mutation is very low (1/N). Each allele in generation *t* has a chance of e^-1^ = 0.368 of not being in generation *t*+1 (3). Each new allele therefore faces a substantial risk of extinction in each generation. Alleles that are lost by drift typically do so after 2ln(2N) generations, i.e. the allele will be lost faster in smaller populations (4). During the clone tree process, the parasite population size drops very low for several weeks in each generation, as it asexually expands after the single-cell bottleneck. Thus, many of the mutations produced at a molecular level will be lost by a combination of drift and selection before they become visible in the clone tree experiment.

# S2. Statistical tests

All statistical tests were performed using the programming language R. The lowest *P*-value output from R is *P* < 2.2x10^-16^, as quoted in main text.

1. Testing Transition to Transversion (Ts:Tv) ratio against expected ratio for clone tree BPS:

- 45 transitions observed out of 85 mutations
- Proportion of mutations that were transition = 45/85 = 0.529
- Binomial 95% Confidence Intervals for transition proportion = 0.418 - 0.639
- Null hypothesis: transition proportion is equal to 2/6 = one third
- Two-sided exact binomial test: *P* = 2.03x10^-4^

2. Testing BPS distribution between substitution types for observed vs expected datasets:

We used 2x6 contingency tables to compare observed and expected data by Pearson’s Chi-squared test. The expected counts assume all substitution types occur at equal rate, but account for the skewed starting template of the *P. falciparum* genome (80% AT : 20% GC).

a) Clone tree observed vs expected

|  | **A**:**T🡪G**:**C** | **G**:**C🡪A**:**T** | **A**:**T🡪C**:**G** | **A**:**T🡪T**:**A** | **G**:**C🡪T**:**A** | **G**:**C🡪C**:**G** |
| --- | --- | --- | --- | --- | --- | --- |
| **Observed** | 21 | 24 | 6 | 20 | 9 | 5 |
| **Expected** | 22.66666667 | 5.666666667 | 22.66666667 | 22.66666667 | 5.666666667 | 5.666666667 |

χ^2^ = 22.049, *df* = 5, *P* = 0.0005125

b) Clone tree observed vs crosses observed

|  | **A**:**T🡪G**:**C** | **G**:**C🡪A**:**T** | **A**:**T🡪C**:**G** | **A**:**T🡪T**:**A** | **G**:**C🡪T**:**A** | **G**:**C🡪C**:**G** |
| --- | --- | --- | --- | --- | --- | --- |
| **Clone tree** | 21 | 24 | 6 | 20 | 9 | 5 |
| **Crosses** | 8 | 8 | 4 | 6 | 4 | 2 |

χ^2^ = 1.2158, *df* = 5, *P* = 0.9434

c) Combined clone tree + crosses observed vs expected

|  | **A**:**T🡪G**:**C** | **G**:**C🡪A**:**T** | **A**:**T🡪C**:**G** | **A**:**T🡪T**:**A** | **G**:**C🡪T**:**A** | **G**:**C🡪C**:**G** |
| --- | --- | --- | --- | --- | --- | --- |
| **Observed** | 29 | 32 | 10 | 26 | 13 | 7 |
| **Expected** | 31.2 | 7.8 | 31.2 | 31.2 | 7.8 | 7.8 |

χ^2^ = 27.52, *df* = 5, *P* = 4.517x10^-5^

3. Testing for mutational equilibrium

We tested whether the proportions of G/C🡪A/T vs A/T🡪G/C counts observed differed significantly from what would be expected if the genome was at mutational equilibrium, defined as G/C🡪A/T count being equal to A/T🡪G/C count. We used the combined 117 clone tree + crosses BPS dataset:

- 45 G/C🡪A/T and 39 A/T🡪G/C observed (total = 84)
- Proportion of mutations that were G/C🡪A/T = 45/84 = 0.536
- Binomial 95% Confidence Intervals for G/C🡪A/T proportion = 0.424 - 0.645
- Null hypothesis: G/C🡪A/T proportion is equal to 0.5
- Two-sided exact binomial test: *P* = 0.5856

4. Testing distribution of mutations in exons, introns, and non-genic loci for observed vs expected datasets:

We used 2x3 contingency tables to compare observed vs expected data by Pearson’s Chi-squared test. The expected counts assume that the distribution of mutations match the underlying proportions of exonic, intronic, and non-genic DNA sequence in the *P. falciparum* genome, taken as 52.63%, 5.79%, and 41.58%, respectively, based on Gardner, M.J., *et al*. 2002. *Nature* 419: 498–511, ref 2 in the main manuscript.

a) Indel distribution between exons, introns, and non-genic loci; observed vs expected:

|  | **Exon** | **Intron** | **Non-genic** |
| --- | --- | --- | --- |
| **Observed** | 15 | 43 | 106 |
| **Expected** | 86.31617094 | 9.497581055 | 68.18624801 |

χ^2^ = 79.788, *df* = 2, *P* < 2.2x10^-16^

b) BPS distribution between exons, introns, and non-genic loci; observed vs expected:

|  | **Exon** | **Intron** | **Non-genic** |
| --- | --- | --- | --- |
| **Observed** | 48 | 3 | 27 |
| **Expected** | 41.05281301 | 4.517142209 | 32.43004478 |

χ^2^ = 1.3443, *df* = 2, *P* = 0.5106

5. Testing counts of indels divisible by 2 and 3bp in observed and expected datasets:

“Expected ratios” assume random indel size, so ~1/3^rd^ of indels would be divisible by three bp and ~1/2 of indels would be divisible by two bp.

a) Indels divisible by three bp inside of exons, observed vs expected ratios

- 13 exonic indels divisible by 3 and 2 exonic indels indivisible by 3 (total = 15)
- Proportion of exonic indels that were divisible by 3 = 13/15 = 0.867
- Binomial 95% Confidence Intervals for proportion of exonic indels that were divisible by 3bp = 0.595 - 0.983
- Null hypothesis: Proportion of exonic indels that are divisible by 3bp = (1/3)
- Two-sided exact binomial test: *P* = 3.143x10^-5^

b) Indels divisible by three bp outside of exons, observed vs expected ratios

- 10 non-exonic indels divisible by 3 and 139 non-exonic indels indivisible by 3 (total = 149)
- Proportion of non-exonic indels that were divisible by 3 = 10/149 = 0.0671
- Binomial 95% Confidence Intervals for proportion of non-exonic indels that were divisible by 3bp = 0.0327 - 0.120
- Null hypothesis: Proportion of exonic indels that are divisible by 3bp = (1/3)
- Two-sided exact binomial test: *P* = 9.607x10^-15^

c) Indels divisible by two bp inside of exons, observed vs expected ratios

- 8 exonic indels divisible by 2 and 7 exonic indels indivisible by 2 (total = 15)
- Proportion of exonic indels that were divisible by 2 = 8/15 = 0.533
- Binomial 95% Confidence Intervals for proportion of exonic indels that were divisible by 2bp = 0.266 - 0.787
- Null hypothesis: Proportion of exonic indels that are divisible by 2bp = (1/2)
- Two-sided exact binomial test: *P* = 1

d) Indels divisible by two bp outside of exons, observed vs expected ratios

- 130 non-exonic indels divisible by 2 and 19 non-exonic indels indivisible by 2 (total = 149)
- Proportion of non-exonic indels that were divisible by 2 = 130/149 = 0.872
- Binomial 95% Confidence Intervals for proportion of non-exonic indels that were divisible by 2bp = 0.808 - 0.921
- Null hypothesis: Proportion of non-exonic indels that are divisible by 2bp = (1/2)
- Two-sided exact binomial test: *P* < 2.2x10^-16^

6. Significance testing for the difference between mutation rate means in Cambodian field isolates vs long-term laboratory-adapted isolates

We compared mean BPS rate and *var* gene exon 1 recombination rate in the long-term laboratory-adapted isolates (combined data for 3D7, HB3, Dd2 & W2) vs Cambodian field isolates (combined data for KH1‑01 & KH2-01).

Mean BPS/ erythrocytic life cycle (with standard deviation, SD), in the laboratory isolates vs Cambodian isolates: 6.75x10^‑3^ (4.22x10^‑3^) vs 4.96x10^‑3^ (3.64x10^‑3^). The difference is not significant; *P* = 0.43, two-sample Welch t-test.

Mean *var* exon 1 recombination/ erythrocytic life cycle (with SD), in the laboratory isolates vs Cambodian isolates: 1.13x10^‑3^ (2.16x10^‑3^) vs 5.61x10^‑3^ (2.40x10^‑3^). The difference is statistically significant; *P* = 0.0153, two-sample Welch t-test.

We applied Bonferroni correction (5) for multiple comparisons by multiplying the t-test *P*-value (*P*=0.0153 for *var* exon 1 recombination rate) by the number of independent null hypotheses being tested (*m*=2), giving *P*=0.0306, which is quoted in the main text of the paper.

# S3. Back-to-back base pair substitutions

In the KH1-01 clone tree, a “double BPS” (GpT 🡪 ApC) was identified in the large gene *PF3D7_1465800*, annotated as “dynein beta chain, putative” by PlasmoDB. The mutations are in positions 12,171 and 12,172 of the exonic sequence of PF3D7_1465800 in exon 4, corresponding to amino acids 4,057 and 4,058, respectively (listed in Table S3). It seems unlikely that BPS would occur along consecutive nucleotides purely by chance. Instead, they may represent a single ‘mutation event’ that resulted in consecutive base pair mutations, e.g. an error arising during DNA repair. We therefore calculated mutation rates using both ‘separate BPS’ counts, where every BPS is counted regardless of its position in the genome, and ‘clustered BPS’ counts, where substitutions occurring along consecutive nucleotides are counted as single ‘mutation events’. In the main text, we quote figures for BPS spectrum and transition : transversion ratios using the total BPS pool with each BPS counted separately; for mutation rates, we used clustered BPS so consecutive BPS were counted as single ‘mutation events’. This distinction only affects the KH1-01 isolate, and the difference is small.

# References from Supplementary Notes

1. Claessens,A., Hamilton,W.L., Kekre,M., Otto,T.D., Faizullabhoy,A., Rayner,J.C. and Kwiatkowski,D. (2014) Generation of Antigenic Diversity in Plasmodium falciparum by Structured Rearrangement of Var Genes During Mitosis. *PLoS Genet.*, **10**, e1004812.

2. Miles,A., Iqbal,Z., Vauterin,P., Pearson,R., Campino,S., Theron,M., Gould,K., Mead,D., Drury,E., Brien,J.O., *et al.* (2016) Indels, structural variation, and recombination drive genomic diversity in Plasmodium falciparum. *Genome Res.*, **26**, 1288–1299.

3. Hartl,D.L. and Ckark,A.G. (2007) Principles of Population Genetics 4th ed. Sinauer Associates, Inc.

4. Chang,H.H., Daniels,R.F. and Hartl,D.L. (2012) Chapter 1: Population Genetics and Parasite Diversity. In Sibley,L.D., Howlett,B.J., Heitman,J. (eds), *Evolution of Virulence in Eukaryotic Microbes*. Wiley-Blackwell.

5. Bland,J.M. and Altman,D.G. (1995) Multiple significance tests: the Bonferroni method. *BMJ*, **310**, 170.

# Supplementary table legends

**Table S1. AT content in a range of eukaryotic organisms.** The *Plasmodium* genus shows high AT content compared with most other eukaryotes. Phylogenetic relationships of the Apicomplexan parasites and their AT contents are shown in Supplementary Figure S1. Data from NCBI genome browser (<http://www.ncbi.nlm.nih.gov/genome/>), accessed 15/07/2016.

**Table S2. Genome coverage for each subclone.** The table indicates, for each sample, the percentage of the 3D7 reference genome that is covered by at least 5 or 10 reads. As expected, 3D7 samples show slightly higher coverage than non-3D7 samples.

**Table S3. *De novo* BPS identified in all clone trees**

**Table S4. *P*-values of two-sample Welch t-test for pairwise comparisons of adjusted BPS mutation rates.** Only G:C🡪A:T mutations are significantly different from other mutation types. (*P* < 0.05 shaded red.)

**Table S5. BPS identified in the progeny of three experimental genetic crosses: HB3xDd2, HB3x3D7, and 7G8xGB4**

**Table S6. *De novo* indels identified in the 3D7 clone tree**

**Table S7. Amino acids involved in coding indels.** Poly(N) homorepeat was defined as having at least one N in the indel sequence, and three consecutive Ns in the indel sequence or 20bp up or downstream from the indel sequence.

**Table S8. *Var* gene recombinations identified in the KH1-01 and KH2-01 clone trees**. *Var* gene recombinations identified in the 3D7, HB3, Dd2, and W2 clone trees are shown in Claessens *et al*. 2014.

**Table S9. Summary of *var* gene recombination data for all clone trees**

**Table S10. List of indels called using Illumina whole genome sequence data validated by PCR amplification and capillary sequencing**

**Table S11. Primer sequences**
